# Probing the subcutaneous absorption of a PEGylated FUD peptide nanomedicine via in vivo fluorescence imaging

**DOI:** 10.1186/s40580-019-0192-3

**Published:** 2019-07-08

**Authors:** Pawel Zbyszynski, Inger Toraason, Lauren Repp, Glen S. Kwon

**Affiliations:** Division of Pharmaceutical Sciences, School of Pharmacy, University of Wisconsin-Madison, Madison, WI USA

**Keywords:** Renal fibrosis, Fibronectin, FUD/pUR4, Subcutaneous drug absorption

## Abstract

The Functional Upstream Domain (FUD) peptide is a potent inhibitor of fibronectin assembly and a therapeutic candidate for disorders linked with hyperdeposition of fibronectin-modulated ECM proteins. Most recently, experiments involving subcutaneous (s.c.) administration of a PEGylated FUD (PEG-FUD) of 27.5 kDa molecular weight yielded a significant reduction of fibronectin and collagen deposition in a murine model of renal fibrosis. The benefits of FUD PEGylation need to be studied to unlock the full potential of the PEG-FUD platform. This work studies the impact of PEGylating the FUD peptide with differently sized PEG on its absorption from the site of injection following s.c. delivery using non-invasive in vivo fluorescence imaging. The FUD and mFUD (control) peptides and their 10 kDa, 20 kDa, and 40 kDa PEG conjugates were labeled with the sulfo-Cy5 fluorophore. Isothermal titration calorimetry (ITC) and confocal fluorescence microscopy experiments verified FUD and PEG-FUD fibronectin binding activity preservation following sulfo-Cy5 labeling. Fluorescence in vivo imaging experiments revealed a linear relationship between the absorption apparent half-life (t_1/2_) and the MW of FUD, mFUD, and their PEG conjugates. Detected drug signal in the kidney and bladder regions of mice suggests that smaller peptides of both the FUD and mFUD series enter the kidney earlier and in higher amounts than their larger PEG conjugates. This work highlights an important delayed dose absorption enhancement that MW modification via PEGylation can contribute to a drug when combined with the subcutaneous route of delivery.

**Electronic supplementary material:**

The online version of this article (10.1186/s40580-019-0192-3) contains supplementary material, which is available to authorized users.

## Introduction

Renal fibrosis presents a significant clinical challenge that demands development of novel and effective therapeutics. The current standard of care for renal fibrosis involves administration of angiotensin-converting enzyme inhibitors (ACEI) or type 1 angiotensin II receptor blockers (ARBs) that can slow the decline in kidney function but do not revert the morphological damage done to the kidneys [1]. Chronic inflammation and hyperdeposition of collagen are hallmark features of fibrosis. Because of its involvement in both processes [2, 3], fibronectin (FN) has been implicated as a possible therapeutic target for the treatment of fibrotic disorders. Interruption of FN fibril formation and thus ECM deposition can have the downstream effect of modulating attachment of lymphocytes and deposition of collagen and other ECM proteins at the site of injury. The pioneering work of Mosher et al. [4] showed that the terminal 70K region of FN is critical to its ECM assembly. This information was used to engineer a large 6 kDa peptide, the Functional Upstream Domain (FUD) peptide also known as the pUR4 peptide, to have a low nanomolar affinity for the 70K region of FN and thus to have potent FN matrix assembly inhibition activity [5–7]. The FUD peptide was successfully applied in murine models of liver fibrosis, coronary artery disease, and heart failure to reduce the fibrotic burden of each disease [8–10]. The Kwon lab sought to increase the efficacy of this novel therapeutic through modification of FUD at the N-terminus with polyethylene glycol (PEG) of 10 kDa, 20 kDa, and 40 kDa MW, increasing the effective hydrodynamic size of FUD and thereby improving its delivery with a nanotechnology approach. All three variants of this PEG-FUD displayed preserved binding affinity (K_d_) and in vitro FN matrix assembly inhibitory potency of the native FUD peptide [11]. These in vitro and biophysical results complement later successful application of PEG-FUD in a murine model of renal fibrosis.

Efficacy evaluation of a singly 20 kDa PEGylated FUD peptide in the unilateral ureteral obstruction (UUO) murine model of renal fibrosis revealed significant amelioration of renal fibrosis morphological features [12]. Administration of the peptide for seven consecutive days yielded a reduction in levels of fibronectin (~ 70%), collagens I and III (~ 60%), and CD45-expressing cells (~ 50%) in the kidney. Interestingly, PEG-FUD was twice as efficacious in reducing FN content of the diseased kidneys as unmodified FUD. This efficacy improvement is perhaps due to an enhanced plasma exposure and thus therapeutic window that is typical of PEGylated drugs. It is well understood that PEGylation using a sufficiently large PEG moiety can result in a reduction of renal clearance and proteolytic degradation of a drug [13–15]. This is true because a PEG moiety with a hydrodynamic radius of sufficiently large size can significantly reduce the drug’s renal sieving coefficient and can also sterically hinder the interaction of proteolytic enzymes with the drug. Furthermore, PEGylation can provide additional therapeutic enhancements if coupled with the subcutaneous (s.c.) delivery route. Molecular weight (MW) modulation via PEGylation can reduce the rate of absorption and consequently the rate of systemic release of the drug, further enhancing the drug’s therapeutic window. Because the s.c. route is common and thus therapeutically relevant for the delivery of nanomedicines, understanding this aspect of PEGylated FUD is important to informing future development of this therapeutic platform as well as that of other nanomedicines.

Although much remains to be understood about the mechanism of this effect, the relationship between the rate of protein s.c. absorption and MW has been studied in the past in several animal models [16–18]. Peptides and proteins that are small (< 16–20 kDa) are known to enter circulation from the s.c. site primarily through blood capillary diffusion. Larger biomolecules occupying a greater volume like nanobodies and monoclonal antibodies are excluded from this pathway and instead must traverse through the interstitium before entering the lymphatic system, where they cross lymph capillaries into lymph nodes before ultimately entering blood circulation. As a result, the time to maximum serum concentration (T_max_) of monoclonal antibodies administered subcutaneously is around 3–8 days in humans [17]. The work of Kaminskas et al. [19] explored this concept using interferon (IFN, 19 kDa) and its PEGylated variants, IFN-PEG12 (31 kDa) and IFN-PEG40 (60 kDa). It was found that while IFN shows poor uptake into the lymph (< 1%), approximately 20% and 21% of the injected IFN-PEG12 and IFN-PEG40 dose, respectively, was recovered in the thoracic lymph following s.c. administration. Furthermore, the T_max_ of each drug increased with drug MW, suggesting an inverse relationship between drug s.c. absorption and MW. These results thus support the model of increasing drug MW and thus hydrodynamic radius redirecting the pathway of drug transit into systemic circulation and thus reducing the rate of s.c. absorption.

In contrast to other work tracking a series of different proteins of different MW in the plasma or the lymph [20–22], this work explores the behavior of a parent peptide and its PEG conjugates through direct observation of the remaining quantities of the drug at the site of injection as a function of MW. To accomplish this task, a series of three PEG-FUD conjugates have been synthesized. The FUD and 10 kDa PEG-FUD peptides have a MW (6 kDa, 17 kDa) that lies below the 16–20 kDa capillary diffusion pathway cutoff whereas the 20 kDa and 40 kDa PEG-FUD have a MW (27.5 kDa, 49.5 kDa) that matches the lymphatic flow pathway. The FUD peptide and all three of its PEG conjugates have additionally been attached with a sulfo-Cy5 label to facilitate direct observation of drug levels via in vivo fluorescence imaging. Furthermore, the work was repeated using an analogous peptide with no activity for fibronectin, the mutated FUD peptide termed mFUD. Together, the FUD and mFUD series will provide two case studies demonstrating the effect of size modification via PEGylation on the s.c. absorption of a a nanotherapeutic. This work will illustrate a powerful therapeutic property of PEGylation when coupled with the s.c. delivery route, informing future development of PEG-FUD and converging it with development of nanomedicines in general.

## Methods

### Generation of FUD, mFUD, and 10–40 kDa PEG conjugates

FUD and mFUD were generated using a recombinant peptide synthesis protocol with His-tag removal modifications [23] reported previously. Their PEG conjugates were generated using reductive amination chemistry as described in previous work [11]. This task was accomplished using methoxy-PEG-aldehyde of the 10 kDa, 20 kDa, and 40 kDa size purchased from NOF Corporation (Kawasaki, Japan). The concentration of FUD, mFUD, and their PEG conjugates was obtained from 280 nm absorbance measurements using ε = 2980 L mol^−1^ cm^−1^ and ε = 4470 L mol^−1^ cm^−1^ for FUD and mFUD, respectively.

### Generation of sulfo-Cy5 labeled FUD, mFUD, and 10–40 kDa PEG conjugates

Each peptide was labeled with the sulfo-Cy5 fluorophore by incubating a 2 mg/mL solution of the peptide presented in 20 mM Tris buffer (pH 8) with 1 eq of 10 mg/mL Sulfo-Cy5-NHS (Lumiprobe) stock solution originally dissolved in DMSO. The reaction proceeded at room temperature for 2 h under stirring conditions. The reaction mixture was then dialyzed ON using 20 mM Tris (pH 8) and a 3000 MWCO dialysis membrane to remove the unreacted label. The reaction products were then purified using ion-exchange chromatography by loading the solution onto a HiTrap Q HP anion exchange column (GE Healthcare Life Sciences, USA) initially equilibrated with Buffer A (20 mM Tris, pH 8.0). Upon sample injection, the column was washed with 2 CVs of Buffer A and then the sample was eluted with a 10 CV 100% gradient of Buffer B (1 M NaCl in 20 mM Tris, pH 8.0). The fraction containing singly labeled drug was collected and snap frozen. The concentration of the labeled drug was determined using absorbance measurement at 646 nm and the extinction coefficient of sulfo-Cy5 at that wavelength, ε = 271000 L mol^−1^ cm^−1^.

### RP-HPLC analysis of sulfo-Cy5 conjugates

Analysis of purified FUD, mFUD, and their 10–40 kDa PEG conjugates was facilitated by a Zorbax SB-C8 4.6 × 75 mm column with a 3.5 μm pore size (Agilent) connected to a Prominence UPLC system (Shimadzu). A gradient of water and acetonitrile both containing 0.1% formic acid at a flow rate of 1 mL/min was used to elute the sample. A fluorescence detector set to the excitation and emission wavelength of sulfo-Cy5 (ex: 646 nm, em: 662 nm) was used to detect the labeled peptides.

### Isothermal titration calorimetry (ITC) of sulfo-Cy5 conjugates

Isothermal Titration Calorimetry experiments of FUD, FUD-Cy5, and 20 kDa PEG-FUD-Cy5 were performed using a VP-ITC (MicroCal, LLC) microcalorimeter with a cell volume of 2.2 mL. Both the peptides and the FN were inserted into separate 3000 MWCO dialysis bags and were dialyzed ON simultaneously into the same 2L Phosphate Buffered Saline (PBS, pH 7.4) solution before each experiment. A typical ITC experiment involved titration of a 35 μM peptide solution into a cell filled with 1.4 mL of 2.7 μM human plasma fibronectin (MilliporeSigma) at a temperature of 25 °C. A total of 39 injections (1 × 1, 4 × 4, and 34 × 8 μL) were delivered in 120 s intervals. The first data point was routinely discarded and a peptide into PBS control experiment was subtracted from each run to account for the peptide heat of dilution. Data were fit using a one set of sites model Lavenberg-Marquardt nonlinear regression in Origin 7.0.

### Confocal fluorescence microscopy of sulfo-Cy5 labeled peptides

The binding of sulfo-Cy5 labeled peptides and their PEG conjugates to developed extracellular FN networks of dermal fibroblast was observed using confocal fluorescence microscopy. An AH1F fibroblast suspension containing 60,000 cells in 2% fetal bovine serum (FBS) + Dulbecco’s Modified Eagle’s Medium (DMEM) was added to a 35 mm Glass bottom dish with 20 mm micro-well (Cellvis). The cells were incubated for 2 h at 37 °C with 5% CO_2_ to facilitate spreading and adhesion of cells. Following incubation, 100 μL of either human plasma FN or Alexa Fluor 488 (ThermoFisher) labeled human plasma FN (A488-FN) was then added to each well for a final concentration of 11 μg/mL. The cells were then incubated for 24 h at 37C to facilitate FN matrix formation. The A488-FN was generated from the same stock of FN as the experiment’s using the manufacturer’s protocol for the NHS ester. On the next day, the liquid contents of the well were removed and replaced with 100 μL of a 500 nM peptide treatment. After a 30 min RT incubation, the cells were washed with Hank’s Balanced Salt Solution (HBSS) containing Ca^2+^ and Mg^2+^ three times and then incubated for 5 min with 100 μL of 5 ug/mL Hoechst 33,342 nucleic acid stain (ThermoFisher) in 2% FBS + DMEM. The cells were again washed three times with HBSS containing Ca^2+^ and Mg^2+^ and presented for imaging. Images were captured using an Olympus FV1000 laser scanning confocal microscope and optimized using FV10-ASW software. The following channels were used for image acquisition: Hoechst—405 nm laser; Alexa 488–488 nm laser; Cy5—635 nm laser. The AH1F cells used in this study are human foreskin fibroblasts that have been described previously [24] and used to demonstrate incorporation of Alexa488-fibronectin in the extracellular matrix [25].

### Fluorescence imaging of FUD, mFUD, and 10–40 kDa PEG conjugates subcutaneous absorption

Female nude athymic 8–10 week old, 20–23 g mice were purchased from Envigo (Madison, WI) and were housed in the Wisconsin Institutes for Medical Research (WIMR) animal facility at the University of Wisconsin-Madison with ad libitum access to food and water. Nude mice were chosen as the model animal because their lack of hair simplifies fluorescence imaging experiments. Animals were maintained in humidity and temperature-controlled rooms under 12 h light/dark cycles. All work was conducted under protocol M005844, reviewed and approved by the University of Wisconsin-Madison Institutional Animal Care and Use Committee. A standard drug dose contained 36.2 nmol (12.5 mg/kg of FUD equivalents) of FUD, mFUD, or 10–40 kDa PEG conjugate and 0.552 nmol of its corresponding sulfo-Cy5 conjugate in 100 μL. In other words, each drug dose was spiked with 1.5% of the sulfo-Cy5 labeled drug for a final sulfo-Cy5 labeled drug concentration of 5.52 μM. This concentration was chosen because of previously performed pilot studies and well-plate experiments showing no significant fluorophore signal saturation or quenching activity in that concentration range. The total amount of drug present in each dose was chosen to parallel previous work in the murine UUO renal fibrosis model [12]. A drug dose stock solution was prepared by mixing an appropriate amount of unlabeled and labeled drug in a conical vial. The solution was dialyzed overnight into PBS, pH 7.4 using a 3000 MWCO dialysis membrane. The drug dose stock solution was then reduced in volume to the appropriate drug concentration using a 3000 MWCO Amicon Ultra-4 centrifugal unit. All dose concentrations were verified before injection. All drug doses were filtered using a sterile 0.2 μm syringe filter prior to injection.

**Fig. 1:**
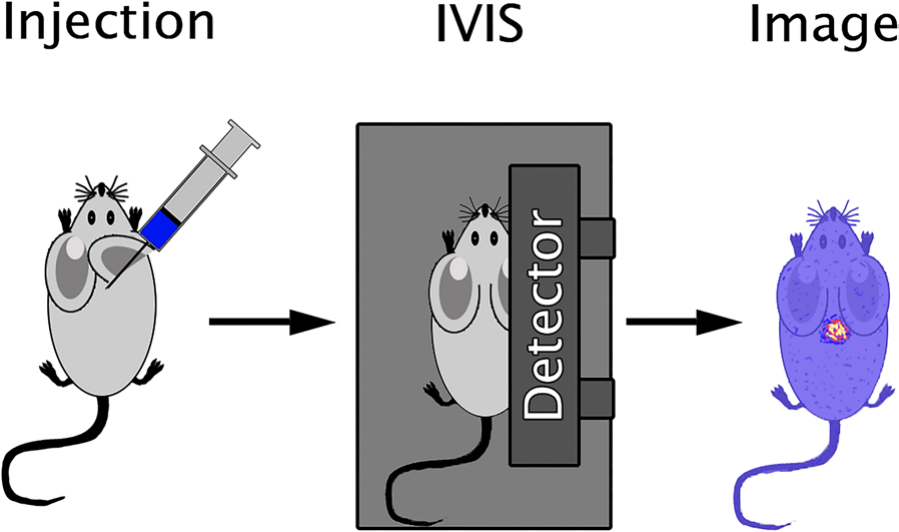
Schematic representation of the in vivo fluorescence imaging experiment. A dose of a sulfo-Cy5 labeled drug is injected subcutaneously between the shoulder blades of the mouse, the animal is imaged using the in vivo imaging system (IVIS), and a 2D fluorescence image of the mouse is produced

Fluorescence imaging of the s.c. site of injection was carried out using the IVIS Spectrum (Perkin Elmer) system using a filter set with an excitation wavelength of 660 nm and an emission wavelength of 680 nm. Identical imaging conditions that included exposure time (1 s), binning (medium), F/Stop (2), and field of view (13 × 13 cm), and order of animal placement were used when acquiring each image of each animal group. The mice were placed under 2% isoflurane anesthesia and were positioned on their abdomen inside of the imaging chamber. Each dose was delivered subcutaneously between the shoulder blades of the animals (n = 3 per treatment group) (Fig. 1). The animals were anesthetized immediately before each session of imaging. Dorsal, ventral, and lateral images of each animal group were taken before drug injection, immediately following injection, and at time points of 30 min and 1, 3, 6, 12, 24, 36, and 48 h. The animals were sacrificed following the last time point. LivingImage (Perkin Elmer) software was used to analyze each view of each animal group. The software’s autodraw feature set to a 5% threshold was used to create unique regions of interest (ROI) around the site of injection on each animal’s dorsal view at t = 0 min for FUD or mFUD and t = 30 min for the PEG conjugates. These initial ROIs were consistently applied to each dorsal image view at each subsequent time point to quantify each drug’s total fluorescence signal in radiant efficiency units. Baseline autofluorescence extracted from the “before injection” views were routinely subtracted from each value. The fraction of drug dose present at the site of injection (F_SC_%) for each drug was determined by dividing the fluorescence signal extracted from the ROI at each time point by the maximal observed ROI fluorescence signal, assuming this value to represent the total drug dose.

The F_SC_% values extracted from each drug series were modeled using nonlinear regression in GraphPad Prism (8.0.2, GraphPad Software, San Diego, California, USA). To accomplish this task, the one phase decay function with a 0 ≤ terminal plateau constraint was applied to the terminal phase of each data series. This function’s fitting model is: F_SC_ = (F_SC,0_ − Plateau) · exp (−k · t) + Plateau, where F_SC,0_ is the dose fraction present at the site of injection at time = 0, Plateau is the value of the signal’s terminal asymptote, and k is the rate constant for the loss of drug from the s.c. site. Because the larger drugs demonstrated a signal reduction lag in the initial time points, the model was applied to the terminal time points of 0–48 h, 1–48 h, 3–48 h, and 6–48 h for FUD and 10 kDa, 20 kDa, and 40 kDa PEG conjugates, respectively. The apparent half-life (t_1/2_) of the drug absorption indicating 50% loss of drug from the s.c. site was calculated from this fitting model using the condition of F_SC_ = 50. The GraphPad Prism software was used to analyze significant differences among F_SC_ values of FUD and mFUD peptides in one MW group as well as a group containing peptides and their immediately larger MW partners. The Student’s t-test with the Holm-Sidak method multiple comparisons correction was used to accomplish this task. Probability (p) ≤ 0.05 was considered to be significant.

## Results and discussion

### Synthesizing and purifying peptides and their sulfo-Cy5 conjugates

The FUD, mFUD, and 10–40 kDa PEG Conjugate peptides were synthesized and labeled with sulfo-Cy5 to support later in vivo fluorescence imaging experiments.

#### Sulfo-Cy5 conjugate synthesis

The sulfo-Cy5 labeled native and PEGylated peptides were successfully synthesized via coupling of the NHS reactive group of the sulfo-Cy5 fluorophore and the primary amines of the Lysine residues and the N-terminus of the peptides. This reaction chemistry is nonspecific at the pH 8 reaction conditions used in this synthesis. Following incubation of the drug and the label, the intensely blue solution was dialyzed using pH 8 Tris. The previously clear dialysate gained a slight blue color, indicating that unreacted dye had passed from the reaction products into the dialysate. This observation suggests that some unlabeled peptide remained in solution as the sulfo-Cy5 and the drug were added in equimolar quantities. Following dialysis, the synthesis products were purified using ion-exchange chromatography (IEX).

**Fig. 2:**
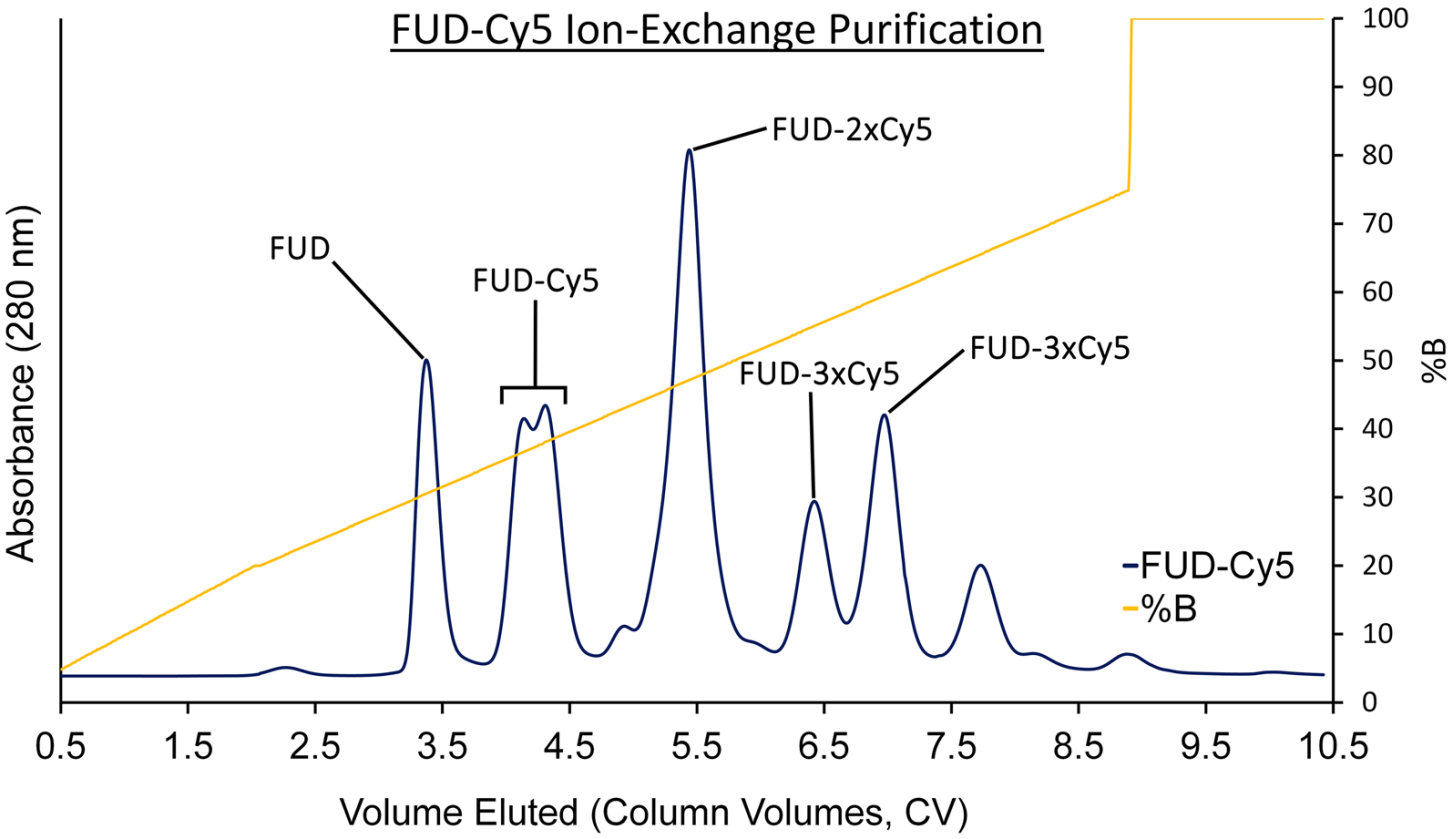
Ion-exchange (IEX) chromatogram showing separation of singly labeled FUD-Cy5 from unreacted and multiply labeled FUD. The sample was loaded onto an anionic exchanger and eluted via application of a mobile phase gradient containing 20 mM Tris (pH 8) in the A side and 1 M NaCl in 20 mM Tris Buffer (pH 8) in the B side

Singly sulfo-Cy5 labeled FUD and its 10–40 kDa PEG conjugates were successfully isolated from other reaction products components using IEX with an anionic exchanger. As shown in Fig. 2, the IEX chromatogram of FUD contains a distribution of peaks that correspond to an increasing charge of the analyte. This pattern is representative of increased degree of product labeling because each sulfo-Cy5 molecule contains two negatively charged sulfone groups. Each major resolved peak was fractionated and the identity of each analyte was determined using UPLC-ESI ultra high resolution QTOF mass spectrometry (data not shown). The first five elution peaks of the FUD-Cy5 reaction products IEX chromatogram corresponded to unreacted FUD (single peak, 30.5%B), singly labeled FUD-Cy5 (split peak, 36.7%B and 38.0%B), doubly labeled FUD-Cy5 (single peak, 47.1%B) and triply labeled FUD-Cy5 (two peaks, 55.0%B and 59.5%B). The doublet eluting at 36.7%B and 38.0%B was collected, pooled, and treated as the FUD-Cy5 peptide stock solution in the experiments that followed.

**Fig. 3:**
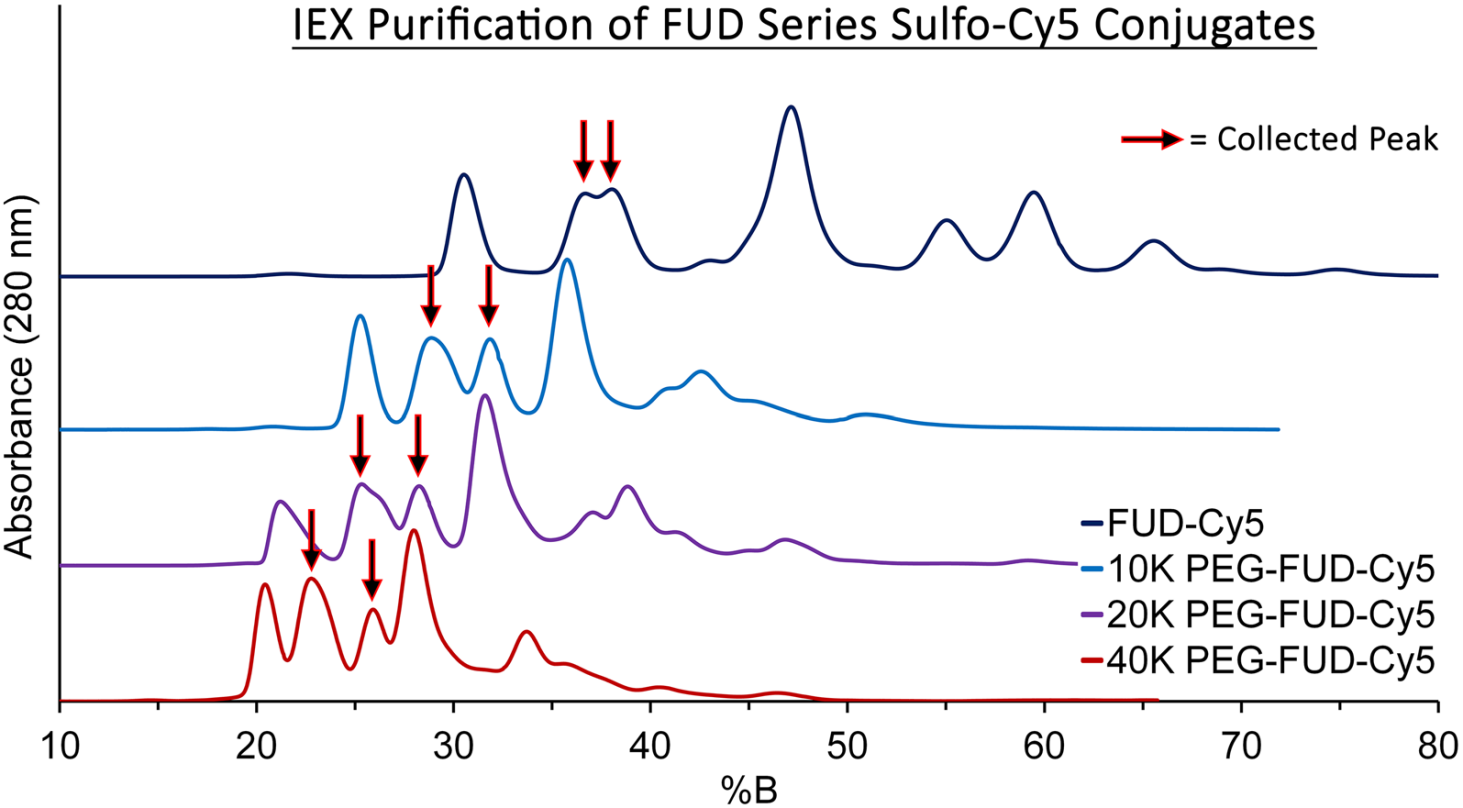
Overlay of ion exchange chromatograms showing the separation of singly sulfo-Cy5 labeled FUD and 10–40 kDa PEG-FUD from the unreacted and multiply labeled peptides. The collected fraction containing the singly labeled drug is indicated with arrows. An anionic exchanger in conjunction with 20 mM Tris (pH 8) A side and 1 M NaCl in 20 mM Tris Buffer (pH 8) B side mobile phases were used to elute the peptides

Purification of labeled 10–40 kDa PEG-FUD peptides via IEX revealed a similar distribution of peaks. Consistently with previous observations in which PEG-FUD showed shorter elution times than FUD [11], increasingly larger PEG-FUD-Cy5 conjugates had increasingly reduced elution times when compared to corresponding peaks of FUD-Cy5 conjugates that were not PEG modified. The same methodology that involved collection of the middle doublet eluting between the first peak and the fourth central peak was used to isolate singly labeled sulfo-Cy5 and PEG conjugated peptides. The collected peaks eluted at 28.9%B and 31.9%B for 10 kDa PEG-FUD-Cy5, 25.3%B and 28.2%B for 20 kDa PEG-FUD-Cy5, and 22.8%B and 25.9%B for 40 kDa PEG-FUD-Cy5. A summary of the IEX chromatograms for all four labeled peptides is presented in Fig. 3. The same synthesis and processing conditions and methodology were also used to purify and collect singly labeled mFUD and 10–40 kDa PEG-mFUD sulfo-Cy5 conjugates. The collected peaks eluted at 37.4%B and 39.3%B for mFUD-Cy5, 29.1%B and 32.2%B for 10 kDa PEG-mFUD-Cy5, 25.5%B and 28.7%B for 20 kDa PEG-mFUD-Cy5, and 21.9%B and 25.4%B for 40 kDa PEG-mFUD-Cy5 (Additional file 1: Fig. S1). The peaks were fractionated, pooled, and used as labeled peptide stock solutions for remaining experiments.

#### Sulfo-Cy5 conjugate chromatographic characterization

**Fig. 4:**
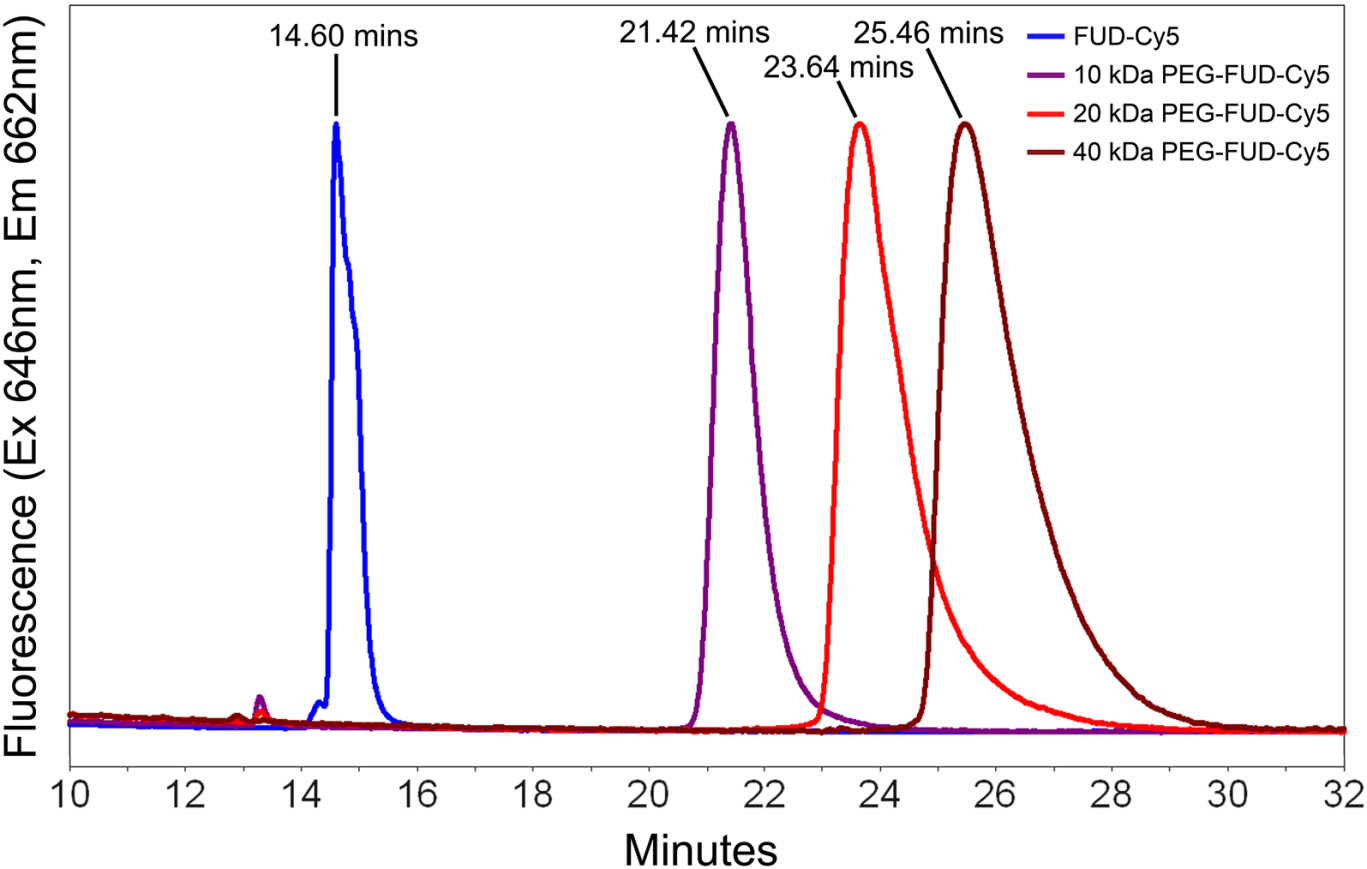
Overlay of reversed phase high performance liquid chromatography (RP-HPLC) chromatograms showing fluorescence activity and preservation of relative retention times of sulfo-Cy5 labeled FUD and its sulfo-Cy5 labeled 10–40 kDa PEG conjugates. The analysis was made using a C8 column and an elution gradient composed of H_2_O + 0.1% FA in the A side and acetonitrile + 0.1% FA in the B side

Reversed phase high performance liquid chromatography (RP-HPLC) analysis confirms synthesis and isolation of sulfo-Cy5 labeled FUD and 10–40 kDa PEG-FUD. Single peaks with fluorescence activity properties of a sulfo-Cy5 molecule eluted with retention times that are similar to those of their parent peptides, confirming successful sulfo-Cy5 labeling of the peptides and complete removal of the free dye. The 10–40 kDa labeled PEG-FUD-Cy5 displayed characteristic peak broadening that is associated with the polydispersity of PEG. The retention times of FUD-Cy5 and its labeled PEG conjugates was observed to be 14.60 min, 21.42 min, 23.64 min, and 25.46 min for FUD-Cy5 and 10 kDa, 20 kDa, and 40 kDa PEG-FUD-Cy5, respectively (Fig. 4). They correspond to previously reported retention times of 12.800 min, 20.602 min, 21.984 min, and 23.461 min for FUD and 10 kDa, 20 kDa, and 40 kDa PEG-FUD, respectively [11], that were obtained using identical elution conditions. A slight increase in the retention time of each peptide compared to its unlabeled counterpart is most likely due to a column interaction enhancement contributed by the hydrophobic domains of the sulfo-Cy5 label. Analysis of mFUD and its 10–40 kDa PEG conjugates produced similar results and conclusions. Single peaks eluted with retention times of 14.92 min, 21.40 min, 23.45 min, and 25.15 min for mFUD and 10 kDa PEG-mFUD, 20 kDa PEG-mFUD, and 40 kDa PEG-mFUD, respectively (Additional file 2: Fig. S2). These retention times match previously published values of 13.259 min and 21.988 min for mFUD and 20 kDa PEG-mFUD, respectively [11]. Analysis via RP-HPLC thus confirms successful synthesis of pure sulfo-Cy5 labeled FUD, mFUD, and 10–40 kDa PEG conjugate peptides. This information supports later quantitative analysis of peptide binding with fibronectin via isothermal titration calorimetry.

### Probing the fibronectin binding interaction with FUD-Cy5 and 20 kDa PEG-FUD-Cy5

The interaction of FUD-Cy5 and 20 kDa PEG-FUD-Cy5 with human plasma FN was studied to determine whether its strength is reduced by chemical derivatization of the drug with sulfo-Cy5.

#### Isothermal titration calorimetry

**Fig. 5:**
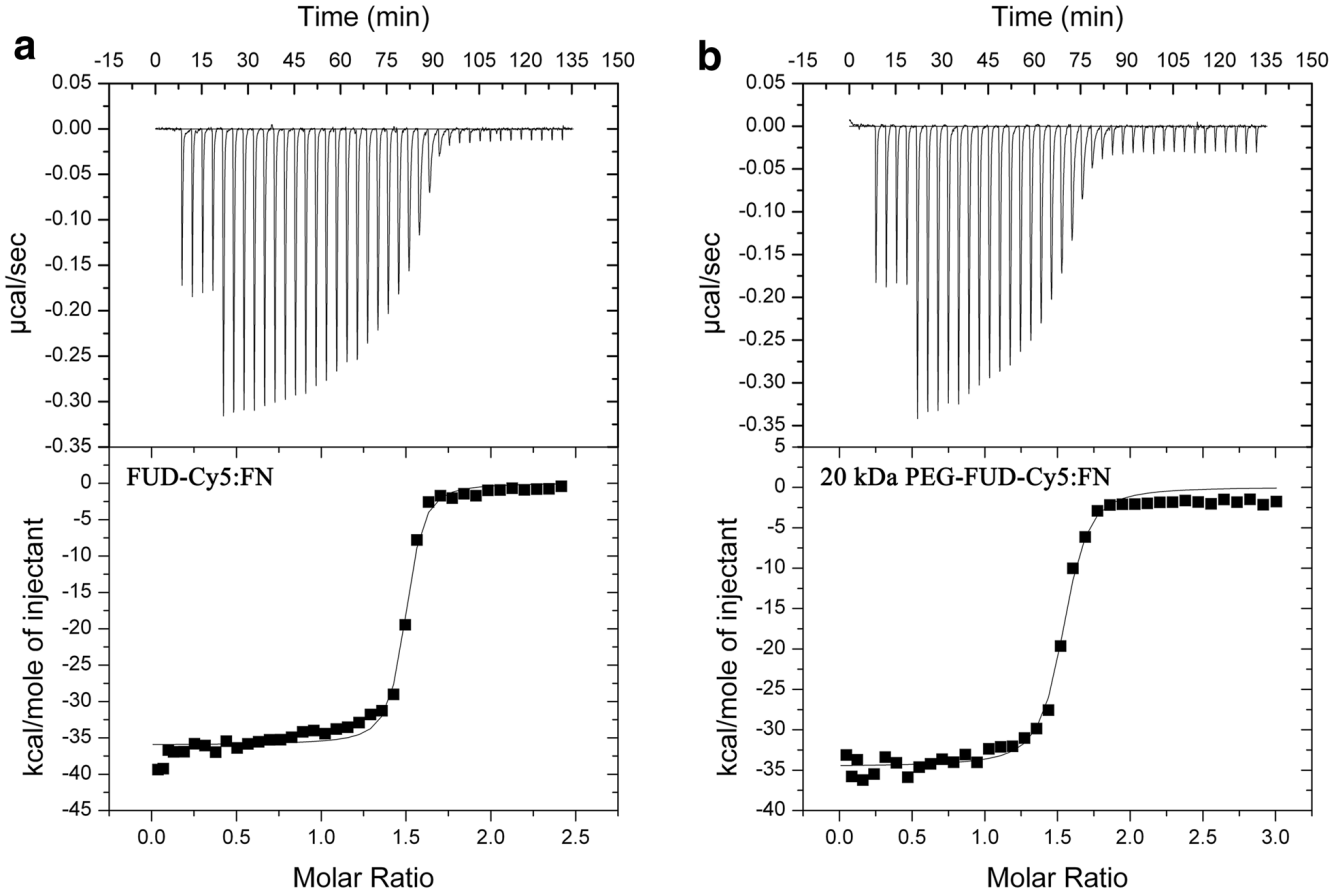
Isothermal titration calorimetry (ITC) was used to determine the binding affinity (K_d_) and other binding parameters of the interaction between the sulfo-Cy5 labeled peptide and human plasma fibronectin. Shown here are isotherms and thermographs of experiments involving **a** FUD-Cy5 or **b** 20 kDa PEG-FUD-Cy5 interaction with FN. All experiments were performed in duplicates using pH 7.4 PBS at 25 °C

The binding of FUD-Cy5 and 20 kDa PEG-FUD-Cy5 with FN was characterized using isothermal titration calorimetry (ITC). There was no significant change in the interaction of human plasma FN with the peptides following their sulfo-Cy5 derivatization. In a typical ITC experiment, a peptide solution was injected into a chamber containing human plasma FN in PBS (pH 7.4) to produce the experiment’s isotherm and thermograph. The binding constant (K_d_) as well as the thermodynamic parameters of the interaction between sulfo-Cy5 labeled peptides and human plasma fibronectin was then extracted from these plots. Sample ITC isotherms and thermographs are presented in Fig. 5. The extracted thermodynamic parameters of each sulfo-Cy5 peptide-FN interaction as well as the extracted binding affinity constants are summarized in Table 1. A K_d_ of 4.7 (± 0.1) nM and 13 (± 2) nM was detected for FUD-Cy5 and 20 kDa PEG-FUD-Cy5, respectively. These values correspond to previously published values of 6 (± 3) nM and 10 (± 2) nM for FUD and 20 kDa PEG-FUD, respectively [11]. Similar enthalpy change of − 35.8 (± 0.2) kcal/mol and − 35 (± 1) kcal/mol and entropy change of − 82.1 (± 0.5) cal/mol and − 82 (± 4) cal/mol were detected for FUD-Cy5 and 20 kDa PEG-FUD-Cy5, respectively. These values correspond to previously published FUD and 20 kDa PEG-FUD values of − 31 (± 1) kcal/mol and − 30 (± 1) kcal/mol for enthalpy change and − 65 (± 3) cal/mol and − 66 (± 7) cal/mol for entropy change, respectively [11]. A control experiment involving injection of unlabeled FUD into FN was also performed, producing again similar K_d_ of 4.3 (± 0.1), ∆H of − 33 (± 2), and ∆S of − 71 (± 6). Both the binding affinity (K_d_) and the thermodynamic parameters of the interaction of either peptide with FN thus remained unaffected by the addition of sulfo-Cy5.

**Table 1:** Extracted isothermal titration calorimetry (ITC) experiment binding parameters

Interaction	[FN] μM	[Peptide] μM	n	K_d_ nM	∆G kcal mol^−1^	∆H kcal mol^−1^	∆S cal mol^−1^
FUD-Cy5:FN	2.7	29.2	1.48 (± 0.01)	4.7 (± 0.1)	− 11.37 (± 0.02)	− 35.8 (± 0.2)	− 82.1 (± 0.5)
20 K PEG-FUD-Cy5:FN	2.6	34.2	1.50 (± 0.01)	13. (± 2)	− 10.8 (± 0.1)	− 35. (± 1)	− 82. (± 4)
FUD:FN	2.6–2.7	39.2–41.4	1.47 (± 0.05)	4.3 (± 0.1)	− 11.40 (± 0.02)	− 33. (± 2)	− 71. (± 6)

Literature [11]	[FN] μM	[Peptide] μM	n	K_d_ nM	∆G kcal mol^−1^	∆H kcal mol^−1^	∆S cal mol^−1^
FUD:FN	1.7–1.8	23–29	1.59 (± 0.06)	6. (± 3)	− 11.4 (± 0.3)	− 31. (± 1)	− 65. (± 3)
20 K PEG-FUD:FN	1.0–2.7	25–42	1.63 (± 0.07)	10. (± 2)	− 10. (± 1)	− 30. (± 1)	− 66. (± 7)

The findings of this ITC study reveal an interesting feature of the FUD peptide that has powerful consequences on the peptide’s ability to be used as an imaging agent. The lack of change in FUD-FN binding affinity upon sulfo-Cy5 labeling is perhaps due to the nature of the interaction between FUD and FN. It involves a cooperative binding of FUD residues located along an extensive region of the peptide with six regions of fibronectin, together contributing to a tight, nanomolar avidity [5]. It is possible that the six possible sulfo-Cy5 points of conjugation (five Lysine residues and the N-terminus) reside in FUD domains that are not critical to this interaction, leading to a lack of binding affinity reduction. The sulfo-Cy5 *N*-hydroxysuccinimide ester (NHS) reactive group is also separated from the fluorophore’s body by a C6 tail, possibly also spatially contributing to this effect. Altogether, the lack of binding affinity reduction suggests that sulfo-Cy5 conjugation does not significantly affect each peptide’s ability to bind to FN, that fluorophore-labeled FUD and its PEGylated analogs will retain their FN fibrillogenesis inhibitory potency, and consequently that the labeled peptides will display a similar in vivo therapeutic action. This suggestion is further supported by in vitro fluorescence microscopy experiments of sulfo-Cy5 labeled peptides binding to assembled exogenous human plasma FN matrix.

#### Fluorescence microscopy

**Fig. 6:**
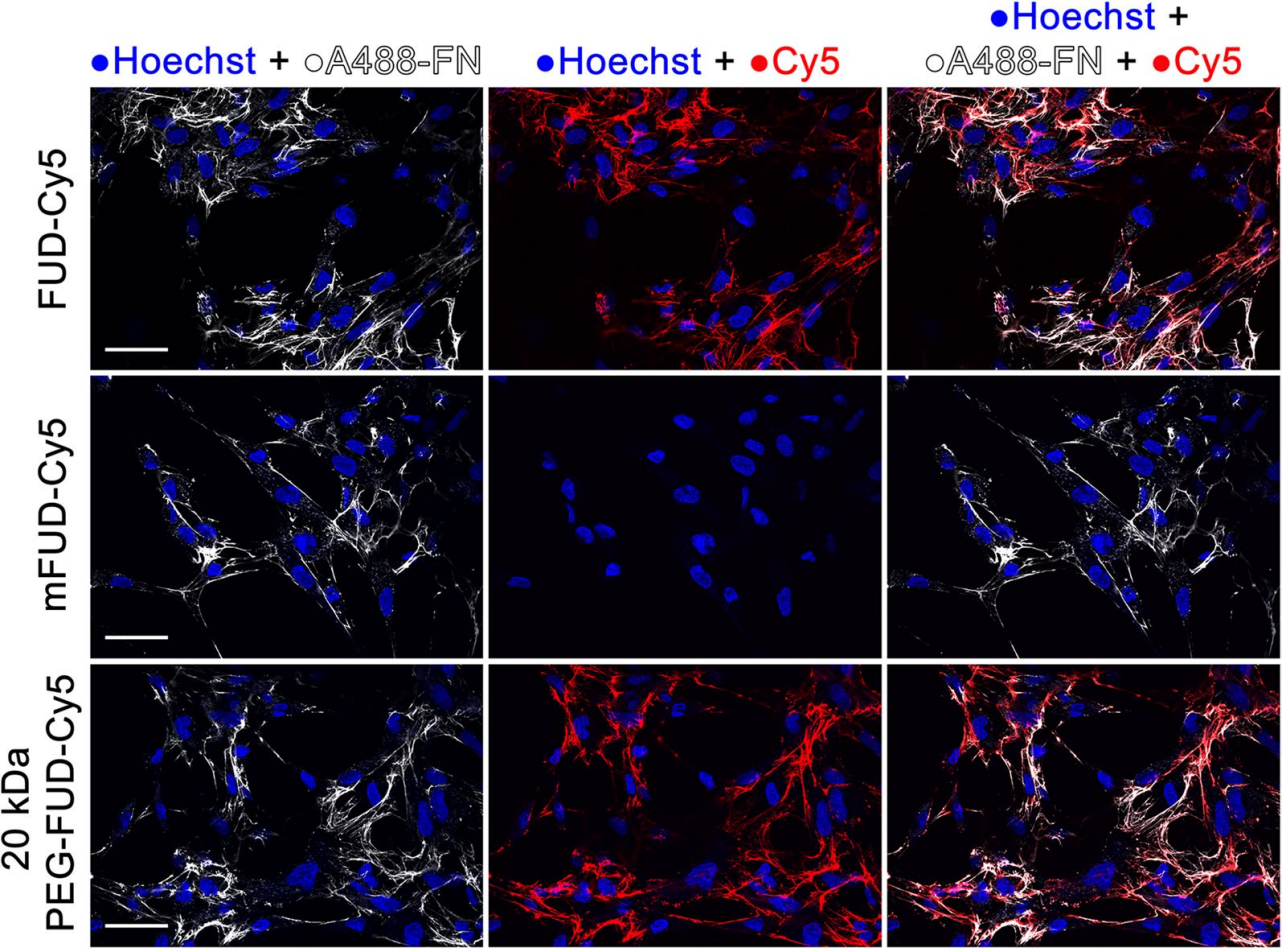
Confocal fluorescence microscopy experiments showing binding of sulfo-Cy5 labeled FUD and 20 kDa PEG-FUD, and lack thereof for mFUD-Cy5, to Alexa Fluor 488 labeled human plasma fibronectin (A488-FN) surfaces assembled by AH1F human foreskin fibroblasts in a glass bottom dish. Each column indicates overlay of listed signal. Overlay of the A488-FN and the peptide-Cy5 signal (last column) reveals colocalization of fibronectin and bound drug. Scale bars = 50 μm

The binding of FUD-Cy5 and 20 kDa PEG-FUD-Cy5, but not mFUD-Cy5, to assembled exogenous human plasma FN was successfully detected in vitro using confocal fluorescence microscopy. The AH1F human foreskin fibroblast cell line known to efficiently assemble rich FN networks from exogenous FN was used for these experiments. Each drug was applied to a glass bottom dish containing a cultured AH1F cell monolayer previously incubated with FN or Alexa Fluor 488 labeled FN (A488-FN). Fluorescence microscopy of the cells produced images containing a multiplexed signal of the Hoechst nuclear stain (405 nm laser), A488-FN (488 nm laser), and the Sulfo-Cy5 labeled peptide (635 nm laser). The captured images are summarized in Fig. 6. Dense FN networks (shown in white) surrounding each cell (nuclei shown in blue) are clearly resolved in each image. A strong sulfo-Cy5 signal (shown in red) was detected in the FUD-Cy5 and 20 kDa PEG-FUD-Cy5 groups, providing evidence of drug binding. This signal is strongly colocalized with the Alexa 488 signal, suggesting that the sulfo-Cy5 labeled peptides are bound specifically to FN. No sulfo-Cy5 signal was detected when the cells were treated with mFUD-Cy5, suggesting that the peptide is not bound to FN. Control experiments were performed to verify that the Alexa 488, sulfo-Cy5, and Hoechst fluorescence signals do not overlap with each other (Additional file 3: Fig. S3). In conclusion, this microscopy study supports the conclusion that FUD-Cy5 and 20 kDa PEG-FUD-Cy5 peptides retain their FN activity and that the mFUD-Cy5 peptide lacks it. Combined with ITC experiments, these two techniques show that the sulfo-Cy5 conjugates of FUD and PEG-FUD retain their tight binding with FN and strong inhibitory activity. This retention of activity validates their use as a tracer molecule surrogate for FUD and PEG-FUD in fluorescence imaging in vivo experiments and highlights their usefulness in visualization of assembled FN surfaces.

### Probing drug s.c. absorption via in vivo fluorescence imaging

The in vivo s.c. absorption and kidney localization of FUD, mFUD, and their PEG conjugates following s.c. injection was studied using IVIS fluorescence imaging.

#### Subcutaneous drug absorption

**Fig. 7:**
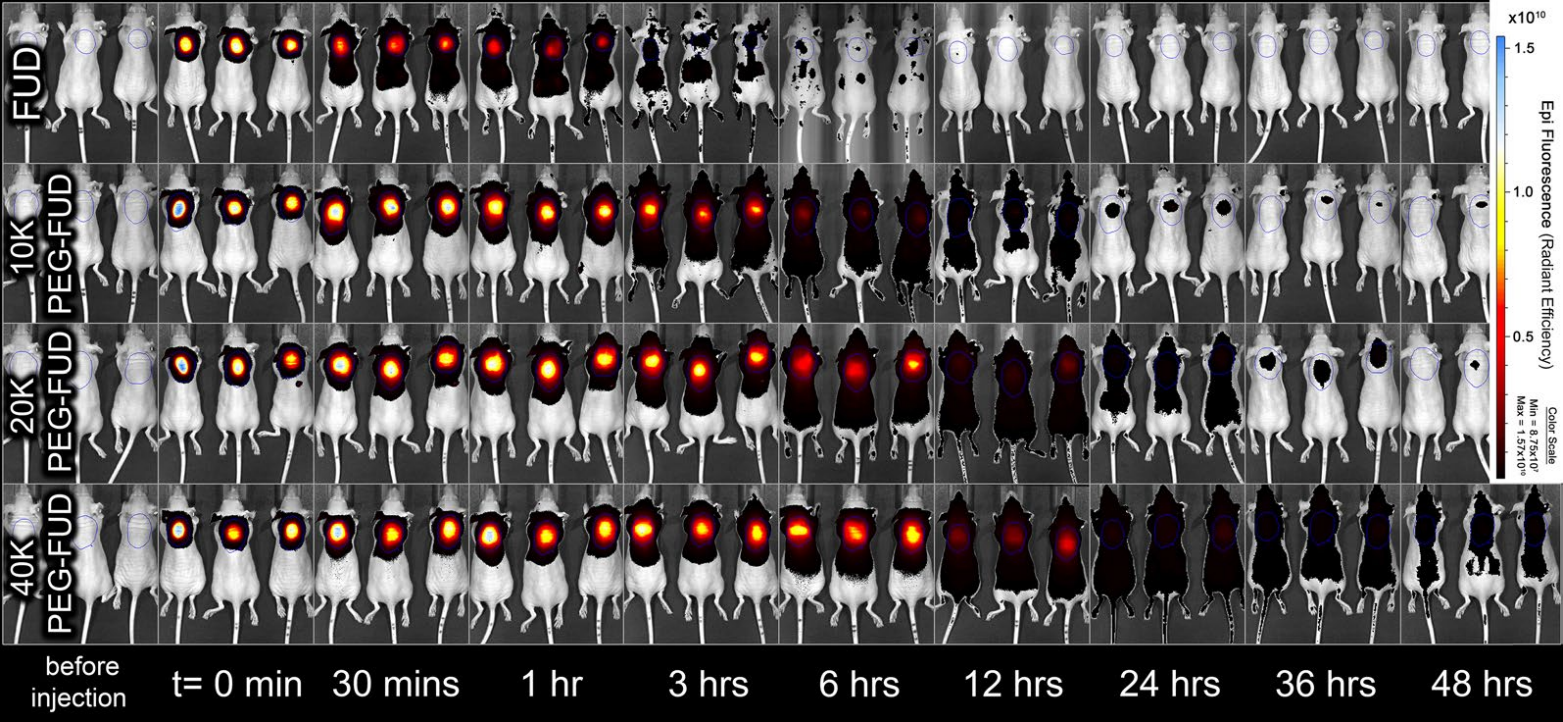
In vivo fluorescence imaging of FUD and 10–40 kDa PEG-FUD remaining dose after s.c. administration of a dose containing the peptide and its sulfo-Cy5 conjugate between the shoulder blades of the mouse. A blue circle drawn between the shoulder blades of each animal indicates regions of interest (ROI) used to indicate the location of the dose to quantify the total remaining dose. Same scale (8.75 × 10^7^–1.57 × 10^10^) is used to visualize the fluorescence intensity in all images

**Fig. 8:**
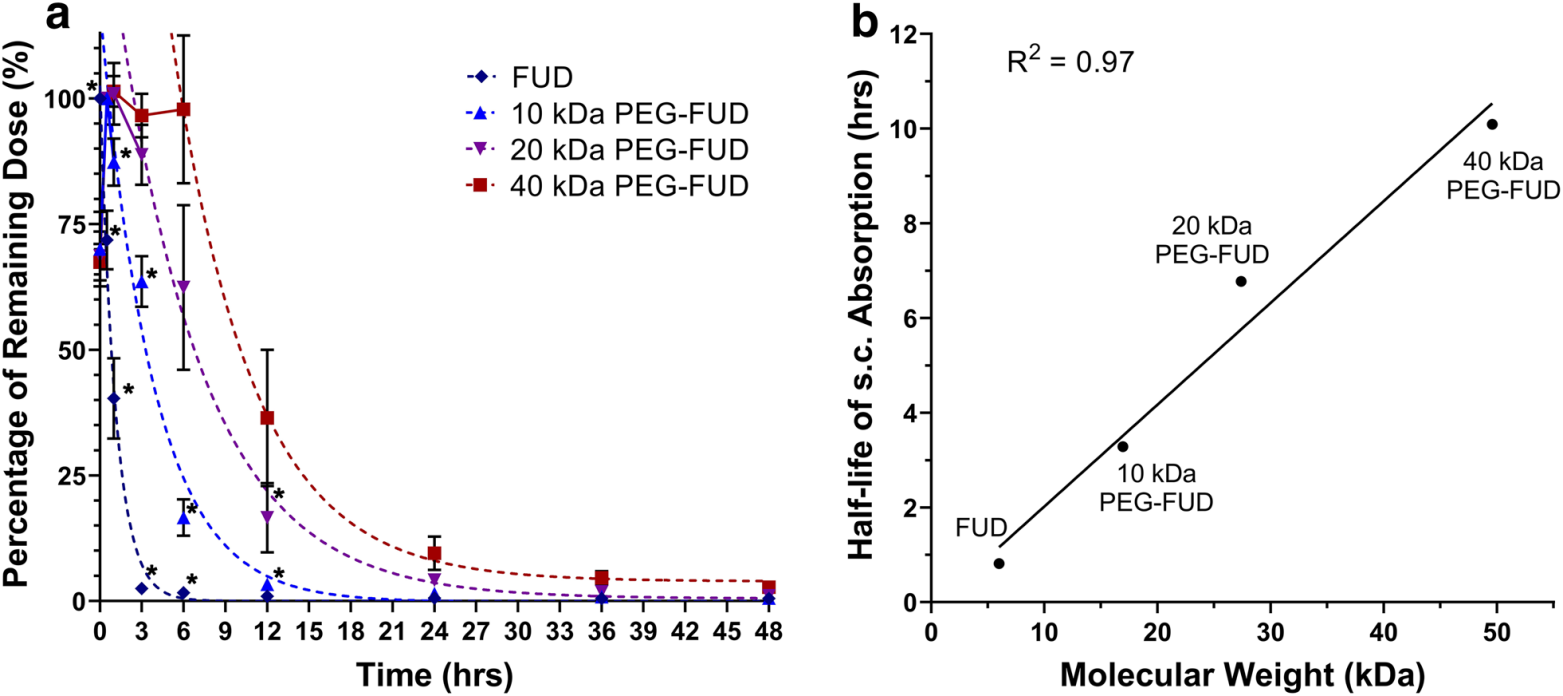
**a** Percentage of drug dose (F_SC_ %) remaining at the site of s.c. injection over time after administration of a FUD or 10 kDa, 20 kDa, or 40 kDa PEG-FUD and a sulfo-Cy5 conjugate dose between the shoulder blades of a mouse. A one phase decay function was applied to the terminal phase of the absorption to calculate F_SC_ % = 50% (t_1/2_) values. Significance (p ≤ 0.05) of difference between mean F_SC_ % values of a peptide and its larger counterpart is denoted with an asterisk (*). n = 3 **b** Relationship between apparent half-life (t_1/2_) of peptide absorption and the peptide’s MW. Linear regression analysis resulted in an r^2^ correlation coefficient of 0.97

A linear inversely proportional relationship was observed between drug molecular weight (MW) and the absorption of FUD, mFUD, and their 10–40 kDa PEG conjugates following s.c. administration. A single drug dose was injected between the shoulder blades of each mouse. Its absorption from the site of injection was observed using noninvasive in vivo fluorescence imaging. The amount of remaining FUD dose rapidly decreased over time, approaching 2.5% of its original value within 3 h. Overlay of fluorescence images provides visual evidence of drug absorption and plasma residence becoming increasingly prolonged with peptides containing a PEG moiety of increasingly larger size (Fig. 7 and Additional file 4: Fig. S4). The fraction of dose remining at the s.c. site (F_SC_) at each time point were calculated for each drug and fit using a one phase decay model (Fig. 8a). The F_SC_ at the 24 h time point was determined to be 0.6 (± 0.1) %, 1.47 (± 0.09) %, 4 (± 1) %, and 10 (± 3) % for FUD and 10 kDa, 20 kDa, and 40 kDa PEG-FUD, respectively. These values are of significant importance because they suggest the possibility of dose carryover when the larger peptides are injected daily and repeatedly in these quantities, as was done previously [12]. The apparent half-life (t_1/2_) of the injected dose reduction for the FUD and the 10 kDa, 20 kDa, and 40 kDa PEG-FUD conjugates was calculated to be 0.81 h, 3.3 h, 6.8 h, and 10.1 h, respectively (Table 2). These values follow a linear relationship with the drug’s MW (R^2^ = 0.97) (Fig. 8b), thus demonstrating that derivatization of FUD with increasingly larger PEG leads to increasingly slower dose absorption from the s.c. site of injection. This experiment was repeated using the mFUD control peptide with no FN activity to test whether binding of FN contributes to this effect.

**Table 2:** Extracted drug s.c. absorption parameters

Peptide	FUD	10K PEG-FUD	20K PEG-FUD	40K PEG-FUD	mFUD	10K PEG-mFUD	20K PEG-mFUD	40K PEG-mFUD
MW (kDa)	6.0	16.9	27.4	49.6	6.0	16.9	27.4	49.6
t_1/2_ (hrs)	0.81	3.28	6.77	10.09	0.86	3.06	5.25	8.36
k (s^−1^)	0.881	0.262	0.160	0.174	0.853	0.323	0.196	0.174
F_SC_, 24 h (%)	0.6 (± 0.1)	1.47 (± 0.09)	4. (± 1)	10. (± 3)	0.00 (± 0.04)	0.43 (± 0.05)	1.9 (± 0.3)	7. (± 2)

**Fig. 9:**
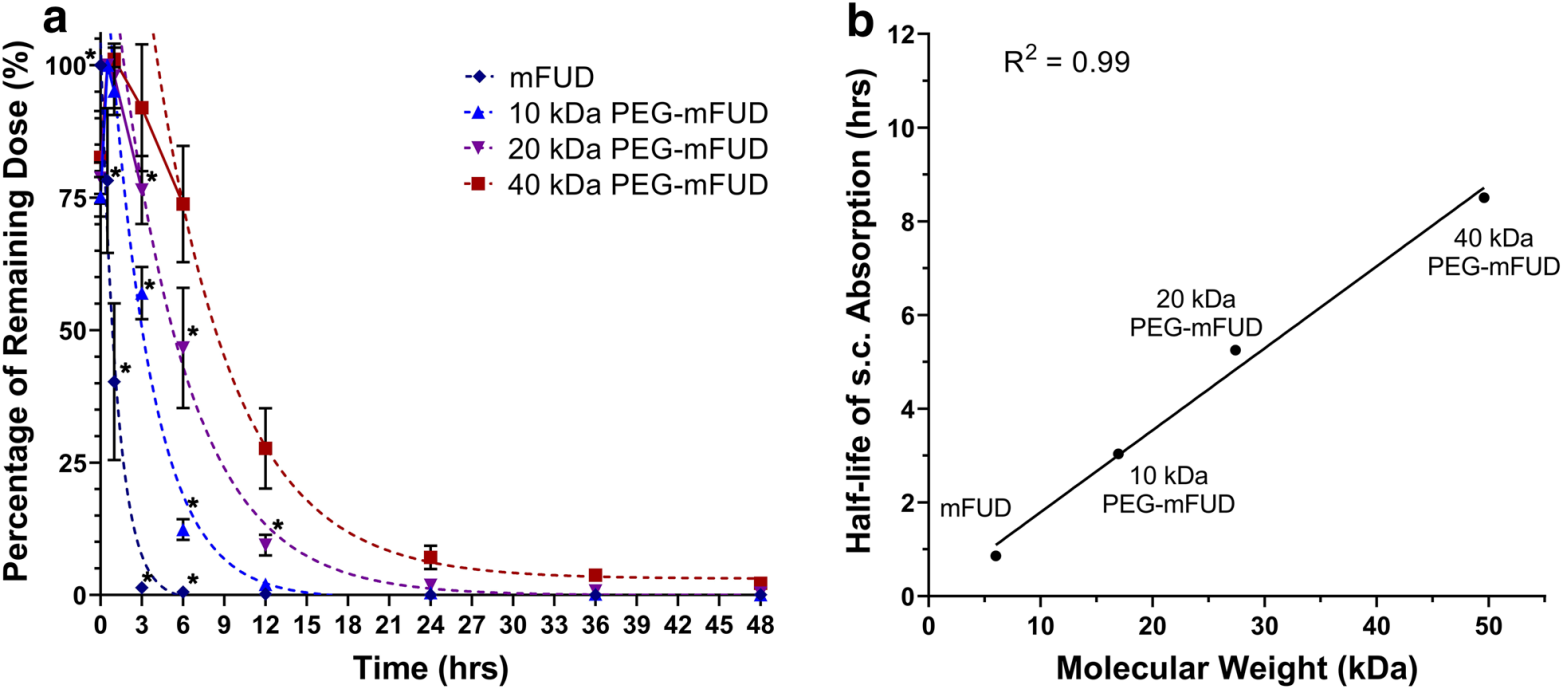
**a** Percentage of drug dose (F_SC_ %) remaining at the site of s.c. injection over time after administration of a mFUD or 10 kDa, 20 kDa, or 40 kDa PEG-mFUD and a sulfo-Cy5 conjugate dose between the shoulder blades of a mouse. A one phase decay function was applied to the terminal phase of the absorption to calculate F_SC_ % = 50% (t_1/2_) values. Significance (p ≤ 0.05) of difference between mean F_SC_ % values of a peptide and its larger counterpart is denoted with an asterisk (*). n = 3 **b** Relationship between apparent half-life (t_1/2_) of peptide absorption and the peptide’s MW. Linear regression analysis resulted in an r^2^ correlation coefficient of 0.99

Experiments with mFUD and its 10–40 kDa conjugates yielded a similar inverse proportionality between the drug MW and its s.c. absorption. The mFUD was absorbed most rapidly of the set (Fig. 9a), with its dose approaching 1.2% of its initial value within 3 h. As was the case with FUD and its PEG conjugates, the F_SC_ of drug doses containing PEG-mFUD peptides of larger MW declined more slowly. After 24 h, approximately 0.00 (± 0.04)%, 0.43 (± 0.05)%, 1.9 (± 0.3)%, and 7 (± 2)% of the original dose was detected at the site of injection for mFUD and 10 kDa, 20 kDa, and 40 kDa PEG-mFUD, respectively (Fig. 9a). The t_1/2_ for each peptide was determined to be of 0.86 h, 3.1 h, 5.3 h, and 8.4 h for mFUD and 10 kDa, 20 kDa, and 40 kDa PEG-mFUD, respectively. Similarly to the FUD series, the t_1/2_ values follow a linear relationship with the drug’s MW (R^2^ = 0.99) (Fig. 9b) The results of the mFUD series thus support the FUD peptide series conclusion of slower s.c. injected drug absorption with larger MW peptides. Furthermore, comparison of F_SC_ means between FUD and mFUD peptides at each MW level revealed statistical significance only in a minority of time points for the FUD/mFUD and 10 kDa PEG-FUD/mFUD comparisons. No statistically significant difference was otherwise observed for the 20 kDa and 40 kDa PEG conjugate sets. This lack of a significant difference suggests that the drug’s binding to FN contributes to the rate of drug absorption of the peptide to a much smaller extent than the physical properties (i.e., higher MW and hydrodynamic volume) conferred onto the peptide by PEGylation.

#### Peptide penetration into the kidney and the bladder

**Fig. 10:**
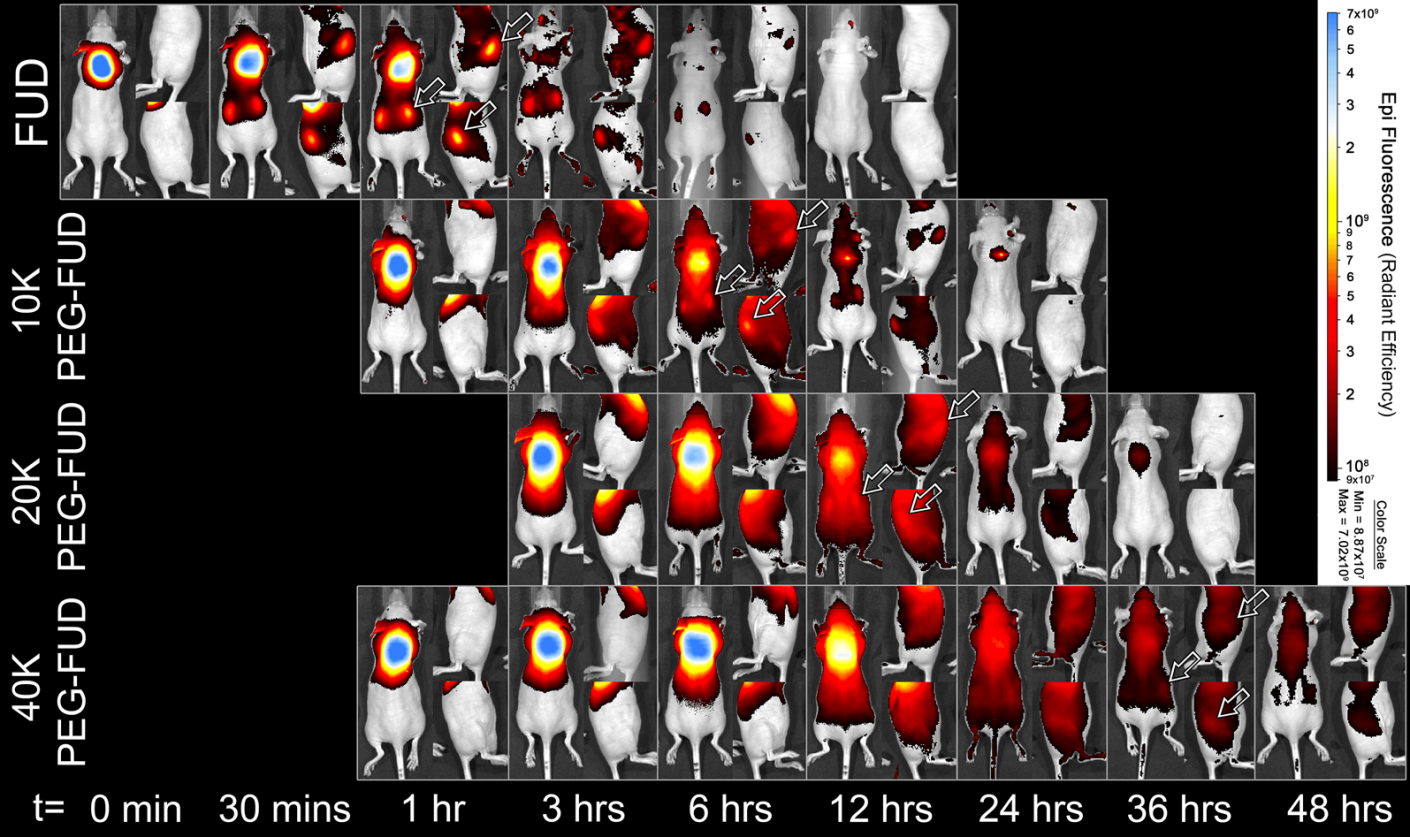
Posterior and lateral views of mice show increased and earlier FUD and PEG-FUD signal in the kidney region for lower MW peptides. A single dose containing a peptide and its sulfo-Cy5 conjugate counterpart was injected s.c. between the shoulder blades of a mouse. Arrows indicate apparent maximal peptide kidney signal

Evaluation of posterior and lateral views of injected animals reveals FUD-Cy5 localization in the kidneys in stronger intensity and at earlier time points than that of 10–40 kDa PEG-FUD-Cy5. FUD-Cy5 signal densification in the kidney region becomes clearly discernable at 30 min, is maximal at 1 h, and can no longer be detected after 12 h (Fig. 10). This state is delayed and less intense for the 10 kDa PEG-FUD-Cy5 signal. The kidney drug signal is first seen at the 3 h mark, is maximal at 6 h, and becomes undetectable at the 24 h time point. This trend continues with 20 kDa PEG-FUD-Cy5. Its signal is seen first very faintly at the 12 h time point and is faintly seen last at the 24 h time point. For 40 kDa PEG-FUD, a very faint kidney signal was observed only at the 36 h time point. The same pattern at the same time points was observed for mFUD and its PEG conjugates as was observed with FUD and its PEG conjugates (Additional file 5: Fig. S5). There appears to be a slightly higher kidney signal intensity for the mFUD-Cy5 and 10 kDa PEG-mFUD-Cy5 compared to FUD and 10 kDa PEG-FUD-Cy5, respectively. This difference is perhaps due to lack of FN binding allowing more of the mFUD peptides to be cleared earlier when the peptides are not sequestered to plasma and ECM fibronectin. Altogether, evaluation of posterior and lateral views of animals show peptides with smaller MW appearing to enter the kidney sooner and in greater abundance than larger PEG-FUD and PEG-mFUD peptides.

**Fig. 11:**
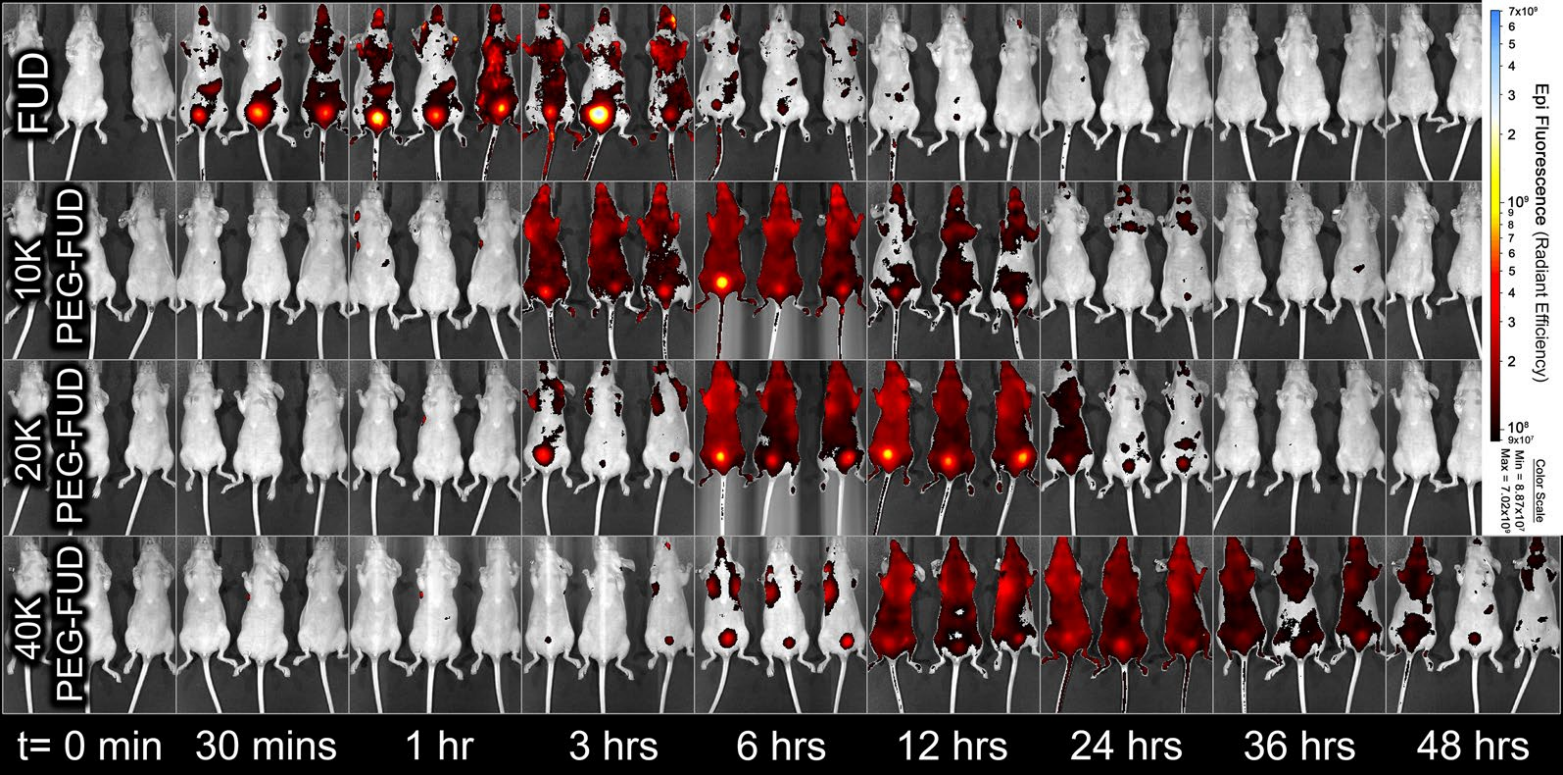
Ventral views of mice show earlier FUD and PEG-FUD signal in the bladder region for lower MW peptides. A single dose containing a peptide and its sulfo-Cy5 conjugate counterpart was injected s.c. between the shoulder blades of a mouse

Evaluation of bladder containing ventral views that show the bladders of injected mice corroborate kidney view conclusions. Labeled drug signal was detected in the bladder region at time points that coincide with time points of signal localization in the kidneys. The FUD-Cy5 peptide signal can be detected in the bladder immediately at the 30 min mark and is continually detected until the 12 h time point (Fig. 11). The 10 kDa PEG-FUD-Cy5 is detected in the bladder at 3–24 h time points. Interestingly, the 20 kDa and 40 kDa PEG-FUD-Cy5 peptides are detected in the bladder at a longer time interval and in larger intensity than expected. The 20 kDa PEG-FUD-Cy5 peptide is first detected in the bladder at 3 h and is last detected at the 24 h time point. The 40 kDa PEG-FUD peptide is detected at the 3–48 h time points, although much more faintly. This observation suggests that despite poor kidney penetration, a small fraction of the 20 kDa and 40 kDa PEG-FUD-Cy5 peptides or their fragments does in fact enter the renal elimination pathway. As before, evaluation of ventral views of mFUD and 10–40 kDa PEG-mFUD injected mice shows a similar pattern at the same time points as was observed for FUD and its PEG conjugates (Additional file 6: Fig. S6). This approach is limited by the mouse’s bladder emptying being controlled by the mouse. It is challenging to discern which time point is truly representative of the maximal bladder signal intensity because it is unclear if the mouse was imaged before it cleared the contents of its bladder. However, because the peptides were indeed detected in the organ, the ventral view data of FUD, mFUD, and their PEG conjugate groups together do reinforce the observation of larger peptides entering renal elimination at a later point following peptide administration.

The observation that FUD and mFUD peptides with increased MW enter the kidney in smaller abundance is consistent with classical theory of glomerular filtration. Although the relationship is complicated by acidity, charge, and geometry of the filtrate [26, 27], it is understood that there exists an inverse relationship between the hydrodynamic volume of a molecule and its ability to participate in glomerular filtration and thus renal elimination. Studies using a variety of model molecules have demonstrated this effect. Most relevantly, a study found renal clearance of an i.v. bolus infused PEG series (6–190 kDa) in a mouse model rapidly declining with MW of 20 kDa or greater [28]. The MW of peptides used in this study is 6 kDa, 16.9 kDa, 27.4 kDa, 49.6 kDa for the parent FUD or mFUD peptides and their PEG conjugates of increasing mass, respectively. The 20 kDa threshold supports this work’s observations as the MW of 10 kDa and 20 kDa PEG-FUD peptides is just smaller and just greater than this threshold. Consequently, the 10 kDa PEG-FUD can be clearly detected in the kidneys while the 20 kDa PEG-FUD is poorly resolved. The 40 kDa PEG-FUD is detected most poorly of the entire set. It is unclear whether the detected signal is of intact peptides or sulfo-Cy5 containing peptide fragments. It is possible that PEGylation also reduces the peptide’s proteolytic degradation in some proportion with MW, amplifying the observed difference in kidney intensity between the peptides of different MW. Whether via renal elimination or other pathways, it is evident that PEGylation has a clear protective impact, decreasing the elimination and increasing the bioavailability of the FUD and mFUD conjugates.

## Conclusions

In this work, we demonstrate that increasing the MW of the FUD peptide through PEGylation reduces its absorption from the site of injection following s.c. administration. The drug’s absorption closely follows a linear inverse relationship with respect to MW, where the lower MW peptide enters circulation faster. This task was accomplished using a sulfo-Cy5 peptide labeling methodology combined with non-invasive in vivo fluorescence imaging. These findings carry exciting implications for the field of fibrosis research, and specifically renal fibrosis research, by revealing a path towards heightened therapeutic accessibility of the parent FUD peptide. Fibronectin inhibition is a possible therapeutic strategy that has already yielded successful results in murine models of liver fibrosis, renal fibrosis, coronary artery disease, and heart failure [8–10, 12]. Understanding that the therapeutic window enhancements provided by PEGylation (i.e., reduction of renal clearance and proteolytic degradation) can be further compounded by delivering the size-optimized PEGylated drug subcutaneously and thereby delaying its absorption and systemic release opens a window of opportunities for therapeutic evaluation of PEG-FUD in models of other pathologies. Idiopathic pulmonary fibrosis is one such pathology that has a high impact, has inadequate standard treatment, and whose progression is dependent on fibronectin activity [29–31]. Increasing the therapeutic window of FUD via PEGylation can also increase its overall therapeutic relevance by reducing the frequency with which the drug would need to be injected by the patient. If the drug is released into systemic circulation more slowly and its plasma levels are maintained to a sufficient level, fewer injections are necessary. An increased therapeutic relevance increases the likelihood that a PEG-FUD therapy is successfully translated from murine models into the clinic.

This work’s insight into PEGylated FUD s.c. delivery intersects with work describing s.c. delivery of other large nanomedicines like drug-conjugated dendrimers and monoclonal antibodies (mAbs). Previous research shows that larger dendrimers display delayed lymphatic drainage, suggesting a MW dependent rate of lymphatic transport [32]. Monoclonal antibodies also consistently release from the s.c. injection site over the course of several days and thus more slowly than smaller drugs [17]. Studying the s.c. absorption of the PEG-FUD and PEG-mFUD model system is generalizable to other nanomedicines and thus complements previous research describing them. As this work’s results are restricted to the murine model, much work remains ahead. There exist significant knowledge gaps in our understanding of the full complexity of macromolecule subcutaneous delivery [17]. It is known that the species-specific, subject-specific, and ECM microenvironment-specific characteristics can have a profound effect on the rate of absorption of a drug [16–18]. The convergence of this work with previous research pertaining to these other nanomedicines will help inform future research supporting nanomedicine clinical development.

This work’s methodology also functions as a case study demonstrating an exciting potential function of the PEG-FUD platform: an imaging agent. In this work, the FUD and PEG-FUD peptides were labeled with sulfo-Cy5 via peptide primary amine (-NH_2_) functionality and the sulfo-Cy5 *N*-hydroxysuccinimide ester (NHS) functionality. Retention of low nanomolar binding affinity for FN following this process suggests that the drug-label conjugate retains its potent fibrillogenesis inhibitory activity in addition to gaining imaging agent properties, and thus can act as a theragnostic agent. A single dose can act as both a fibrosis therapeutic and an imaging agent for use in localizing regions of injury or evaluating disease progression. The NHS functionality is ubiquitous to other labels and is easily accessible, making the labeled PEG-FUD platform generalizable to other technologies as well. One specific example of this technique’s application includes treatment, visualization, and staging the progression of pulmonary fibrosis [29]. The labeled drug or a combination of labeled and unlabeled drug will likely have both therapeutic and imaging action, allowing both disease treatment and visualization and quantification of the FN rich tissue present in the fibrotic lung. A difference in drug signal over time and thus fibronectin content reduction can then be used as a therapeutic endpoint, allowing both treatment and diagnosis of pulmonary fibrosis.

Conclusively, this work presents two important aspects of the PEG-FUD platform that are interesting to explore in the future. The absorption of the PEGylated drug from the s.c. site of administration should be understood in other animal models to bring the drug closer to the clinic. The diagnostic aspect of PEG-FUD should be studied using other tracer labels to probe the drug’s potential as a diagnostic tool for fibronectin-linked pathology applications. We enthusiastically recommend PEG-FUD as a candidate for study in these two areas.

## Additional files

**Additional file 1: Fig. S1.** Overlay of ion exchange chromatograms showing the separation of singly sulfo-Cy5 labeled mFUD and 10-40 kDa PEG-mFUD from the unreacted and multiply labeled peptides. The collected fraction containing the singly labeled drug is indicated with arrows. An anionic exchanger in conjunction with 20 mM Tris (pH 8) A side and 1 M NaCl in 20 mM Tris Buffer (pH 8) B side mobile phases were used to elute the peptides.

**Additional file 2: Fig. S2.** Overlay of Reversed Phase High Performance Liquid Chromatography (RP-HPLC) chromatograms showing fluorescence activity and preservation of relative retention times between sulfo-Cy5 labeled mFUD and its sulfo-Cy5 labeled 10-40 kDa PEG conjugates. The analysis was made using a C8 column and an elution gradient composed of H2O + 0.1% FA in the A side and acetonitrile + 0.1% FA in the B side.

**Additional file 3: Fig. S3.** Fluorescence microscopy control experiments involving a combination of 20 kDa PEG-FUD-Cy5, human plasma fibronectin (FN), Alexa 488 Fluor labeled FN (A488-FN), and Hoechst nuclear stain treatments to AH1F human foreskin fibroblasts grown in a glass bottom dish. Each column indicates overlay of listed signal. The first, second, and third rows verify lack of overlap between the A488-FN, peptide-Cy5, and Hoechst signal. Scale bars = 50 μm.

**Additional file 4: Fig. S4.** In vivo fluorescence imaging of mFUD and 10-40 kDa PEG-mFUD remaining dose after s.c. administration of a solution containing the peptide and its sulfo-Cy5 conjugate between the shoulder blades of the mouse. A blue circle drawn between the shoulder blades of each animal indicates regions of interest (ROI) used to indicate the location of the dose to quantify the total remaining dose. Same scale (8.75x10^7^-1.57x10^10^) is used to visualize the fluorescence intensity in all images.

**Additional file 5: Fig. S5.** Posterior and lateral views of mice show increased and earlier mFUD and PEG-mFUD signal in the kidney region for lower MW peptides. A single dose containing a peptide and its sulfo-Cy5 conjugate counterpart was injected s.c. between the shoulder blades of a mouse. Arrows indicate apparent maximal peptide kidney signal.

**Additional file 6: Fig. S6.** Ventral views of mice show earlier mFUD and PEG-mFUD signal in the bladder region for lower MW peptides. A single dose containing a peptide and its sulfo-Cy5 conjugate counterpart was injected s.c. between the shoulder blades of a mouse.

## Data Availability

The datasets used and/or analyzed during the current study are available from the corresponding author on reasonable request.
