# Supplementary figures and images for: Probing the subcutaneous absorption of a PEGylated FUD peptide nanomedicine via in vivo fluorescence imaging
Source: Nano Converg. 2019 Jul 8;6:22. doi: 10.1186/s40580-019-0192-3 (PMC6612524; doi:10.1186/s40580-019-0192-3)

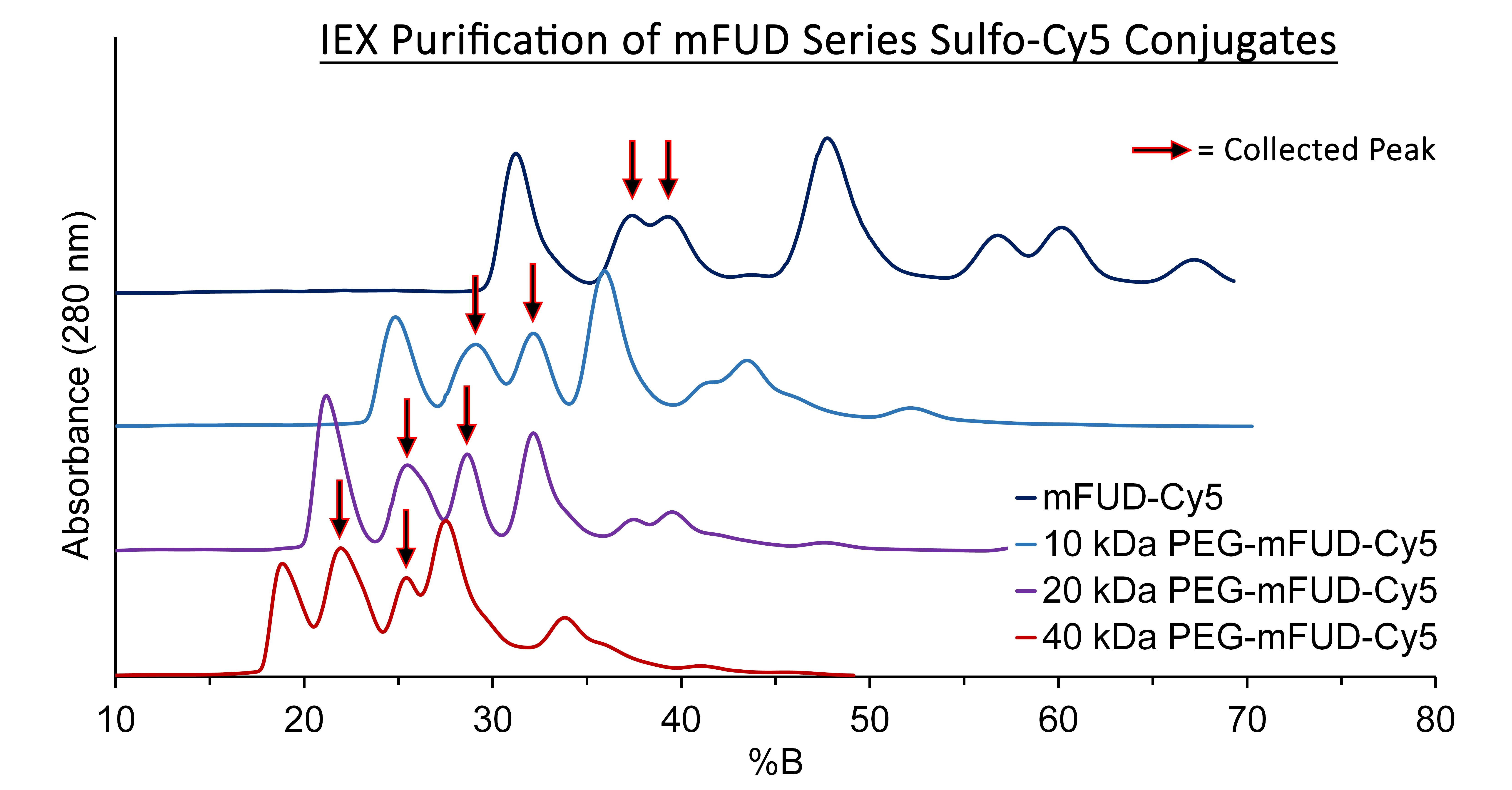

Supplement: Supplementary file 1 — Additional file 1: Fig. S1. Overlay of ion exchange chromatograms showing the separation of singly sulfo-Cy5 labeled mFUD and 10-40 kDa PEG-mFUD from the unreacted and multiply labeled peptides. The collected fraction containing the singly labeled drug is indicated with arrows. An anionic exchanger in conjunction with 20 mM Tris (pH 8) A side and 1 M NaCl in 20 mM Tris Buffer (pH 8) B side mobile phases were used to elute the peptides. [file 40580_2019_192_MOESM1_ESM.jpg]

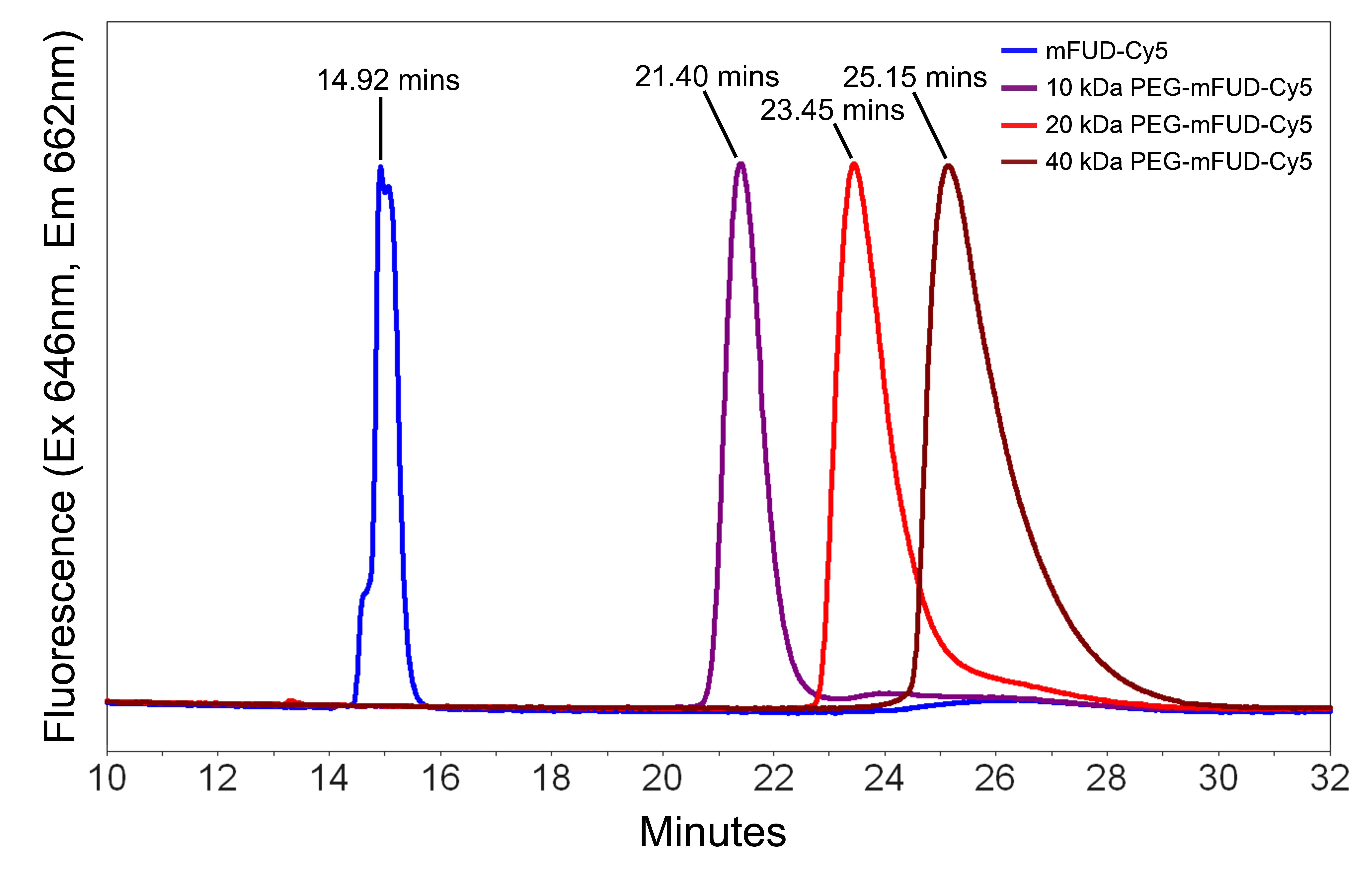

Supplement: Supplementary file 2 — Additional file 2: Fig. S2. Overlay of Reversed Phase High Performance Liquid Chromatography (RP-HPLC) chromatograms showing fluorescence activity and preservation of relative retention times between sulfo-Cy5 labeled mFUD and its sulfo-Cy5 labeled 10-40 kDa PEG conjugates. The analysis was made using a C8 column and an elution gradient composed of H2O + 0.1% FA in the A side and acetonitrile + 0.1% FA in the B side. [file 40580_2019_192_MOESM2_ESM.jpg]

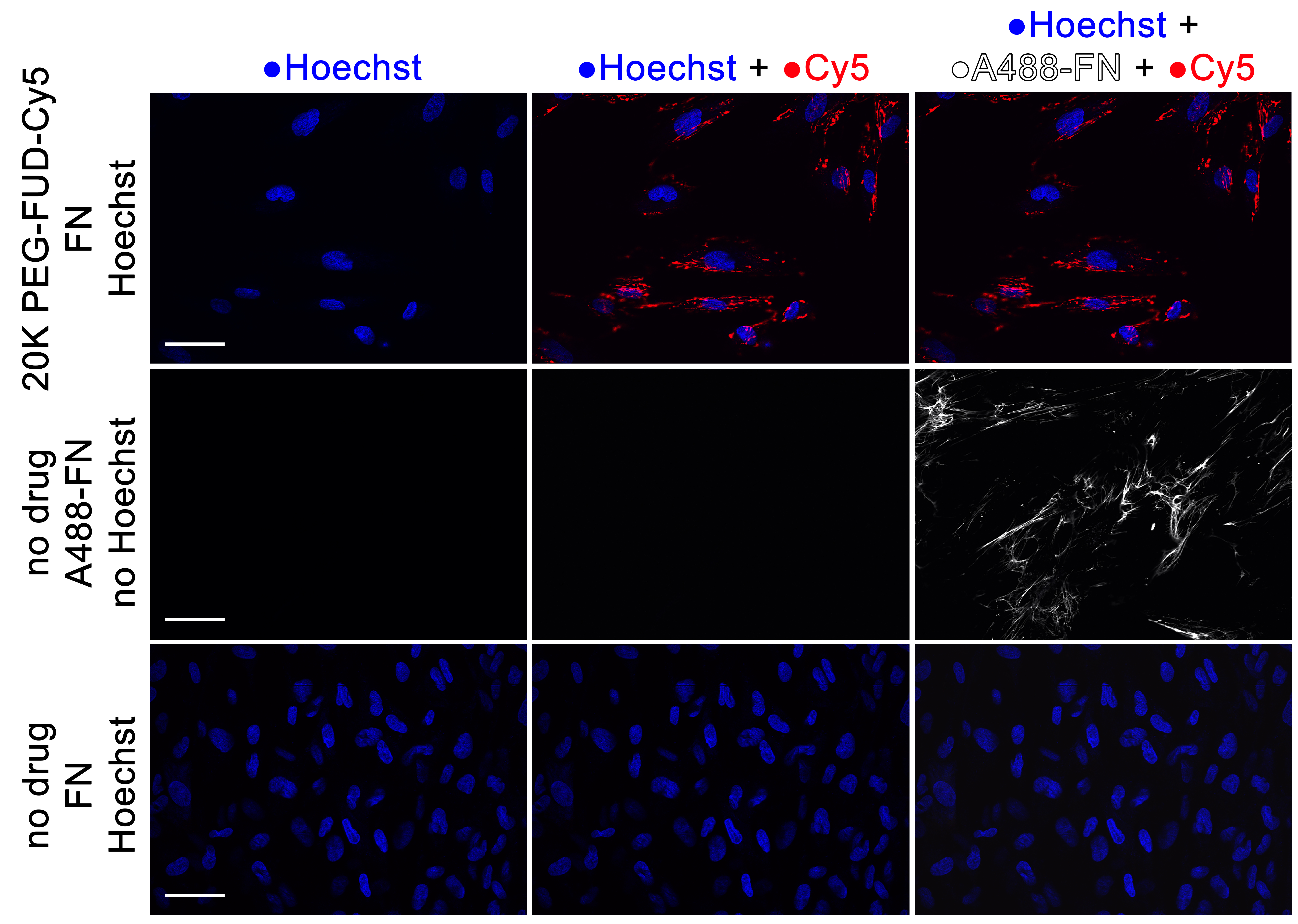

Supplement: Supplementary file 3 — Additional file 3: Fig. S3. Fluorescence microscopy control experiments involving a combination of 20 kDa PEG-FUD-Cy5, human plasma fibronectin (FN), Alexa 488 Fluor labeled FN (A488-FN), and Hoechst nuclear stain treatments to AH1F human foreskin fibroblasts grown in a glass bottom dish. Each column indicates overlay of listed signal. The first, second, and third rows verify lack of overlap between the A488-FN, peptide-Cy5, and Hoechst signal. Scale bars = 50 μm. [file 40580_2019_192_MOESM3_ESM.jpg]

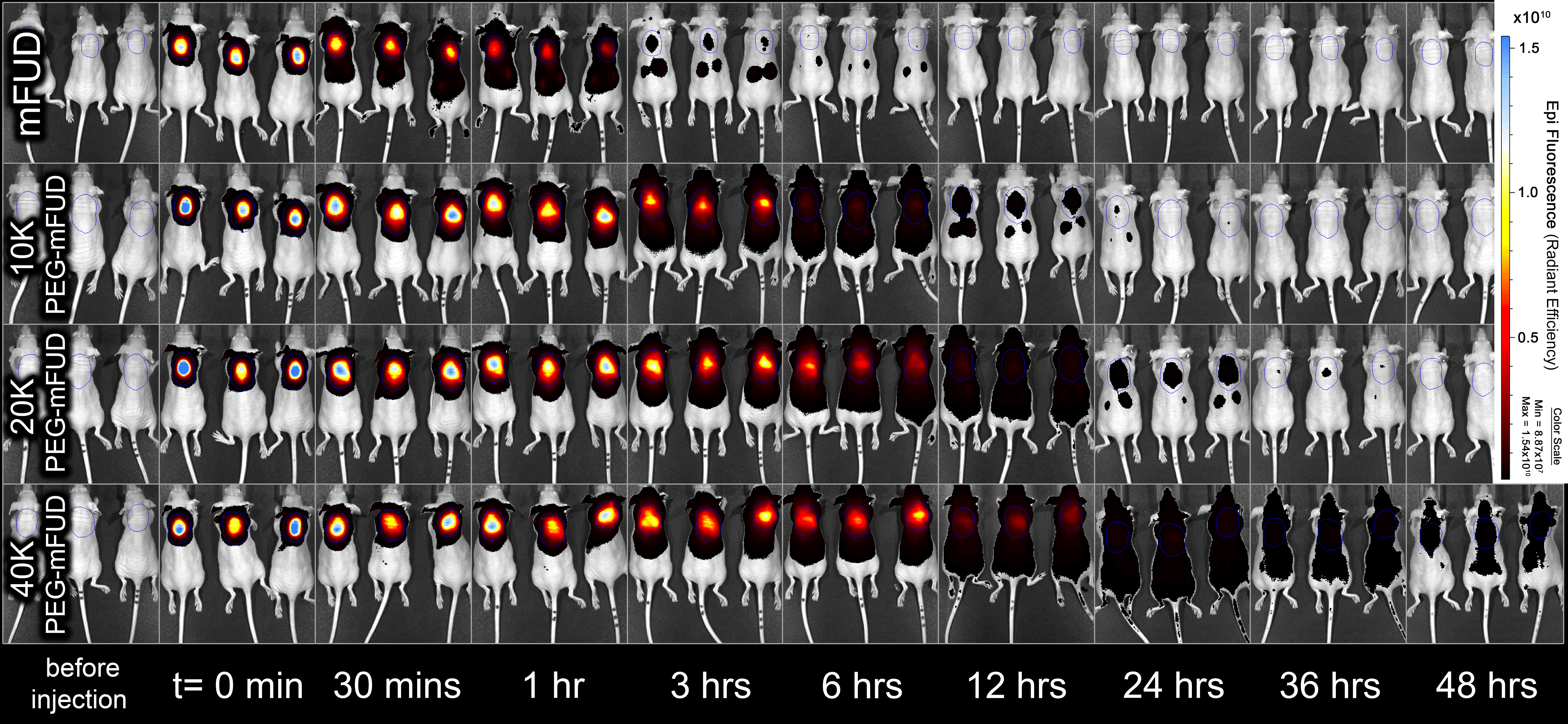

Supplement: Supplementary file 4 — Additional file 4: Fig. S4. In vivo fluorescence imaging of mFUD and 10-40 kDa PEG-mFUD remaining dose after s.c. administration of a solution containing the peptide and its sulfo-Cy5 conjugate between the shoulder blades of the mouse. A blue circle drawn between the shoulder blades of each animal indicates regions of interest (ROI) used to indicate the location of the dose to quantify the total remaining dose. Same scale (8.75x10^7^-1.57x10^10^) is used to visualize the fluorescence intensity in all images. [file 40580_2019_192_MOESM4_ESM.jpg]

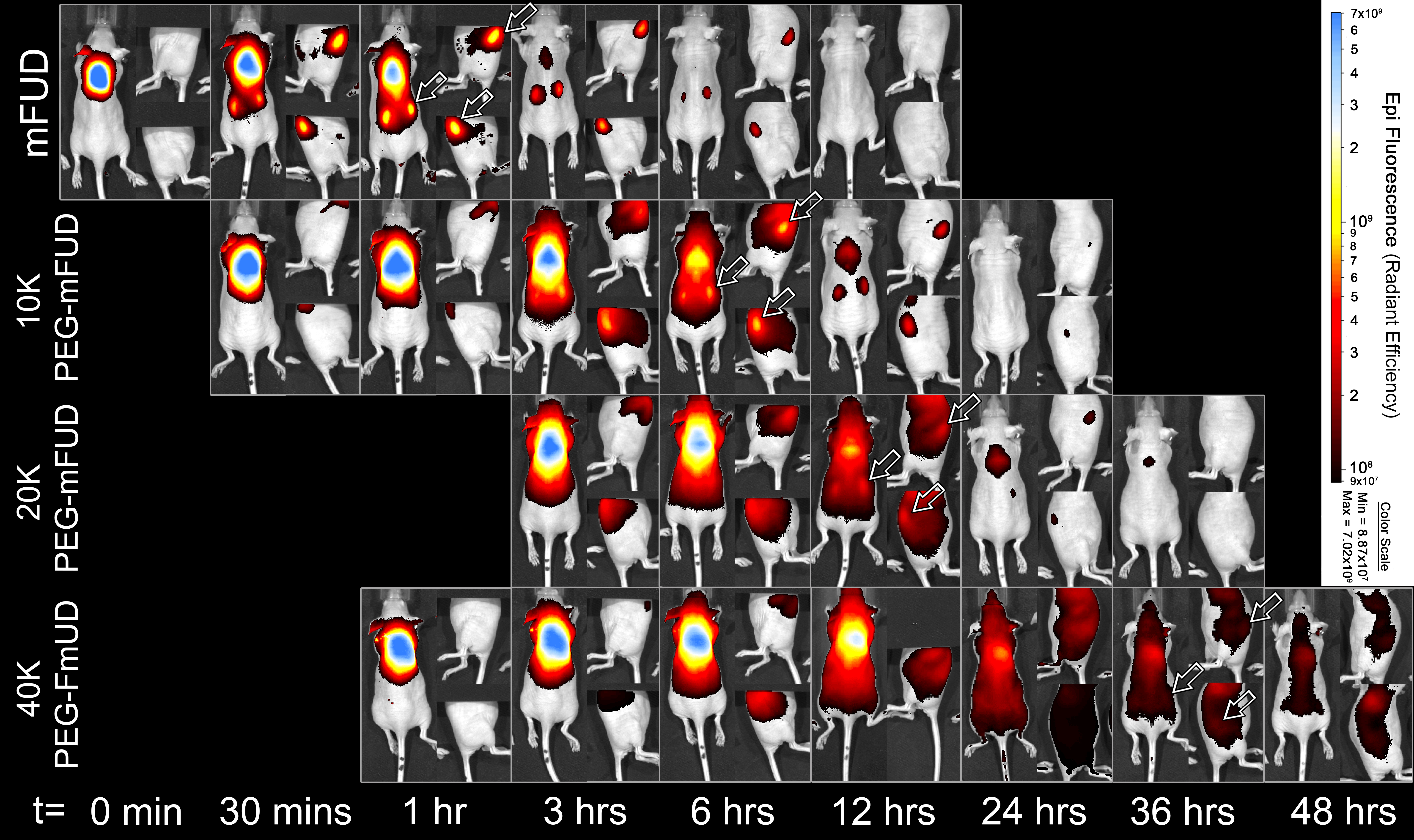

Supplement: Supplementary file 5 — Additional file 5: Fig. S5. Posterior and lateral views of mice show increased and earlier mFUD and PEG-mFUD signal in the kidney region for lower MW peptides. A single dose containing a peptide and its sulfo-Cy5 conjugate counterpart was injected s.c. between the shoulder blades of a mouse. Arrows indicate apparent maximal peptide kidney signal. [file 40580_2019_192_MOESM5_ESM.jpg]

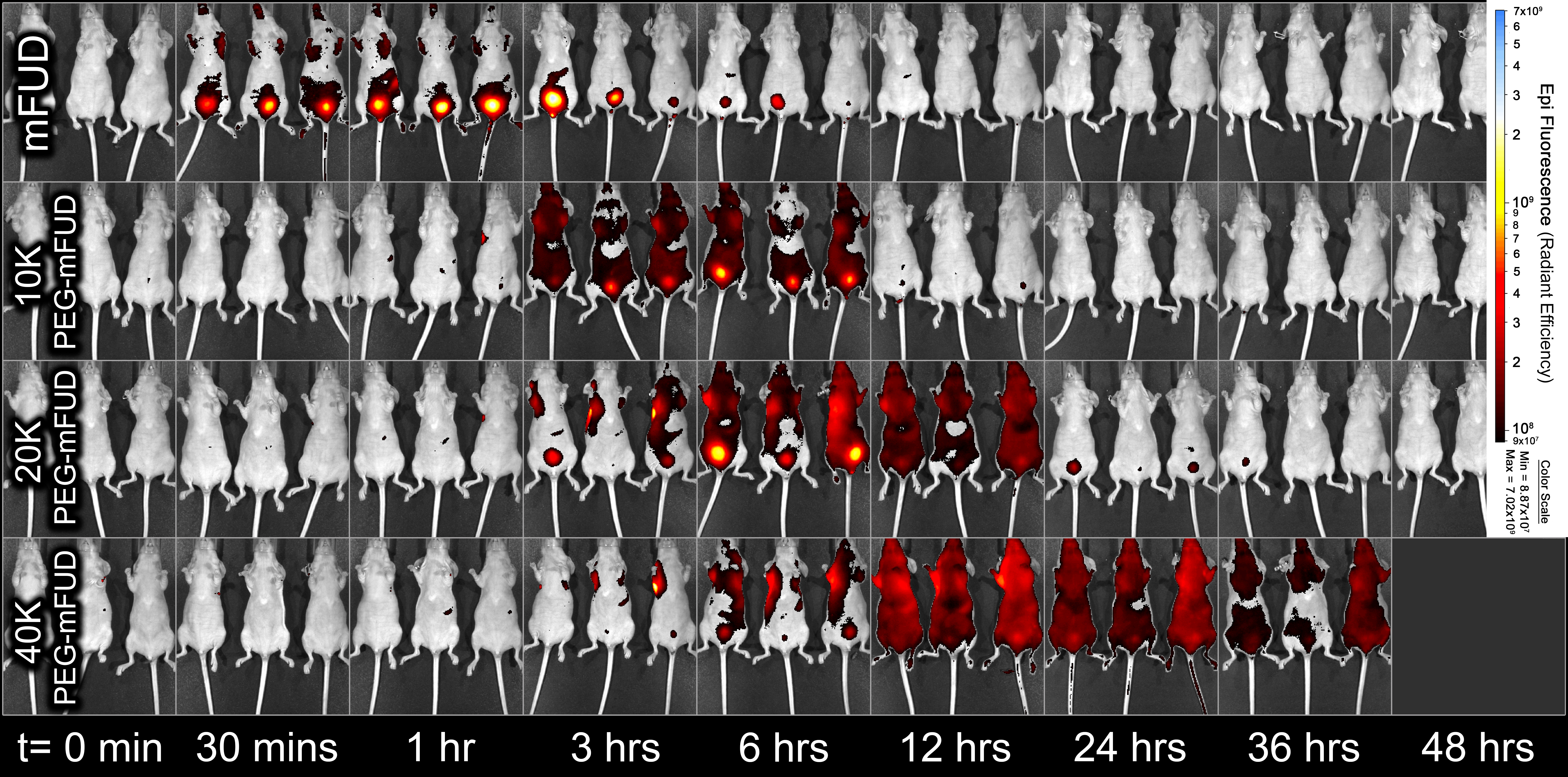

Supplement: Supplementary file 6 — Additional file 6: Fig. S6. Ventral views of mice show earlier mFUD and PEG-mFUD signal in the bladder region for lower MW peptides. A single dose containing a peptide and its sulfo-Cy5 conjugate counterpart was injected s.c. between the shoulder blades of a mouse. [file 40580_2019_192_MOESM6_ESM.jpg]
